# Haplotype-resolved telomere-to-telomere genome of *Aphelenchus avenae* implicates *P5CS* in nematode desiccation stress response

**DOI:** 10.1101/gr.281016.125

**Published:** 2026-04

**Authors:** Yali Zhang, Yayi Zhou, Yangyang Chen, Xueyu Wang, Boyan Hu, Shahid Siddique, Romnick A. Latina, Veronica I. Casey, Dexin Bo, Yucheng Liao, Min Zhang, Ming Sun, Fengjuan Zhang, Dadong Dai

**Affiliations:** 1 State Key Laboratory of Biocatalysis and Enzyme Engineering, Hubei Hongshan Laboratory, School of Life Sciences, Hubei University, Wuhan 430062, China;; 2 National Key Laboratory of Agricultural Microbiology, Huazhong Agricultural University, Wuhan 430070, China;; 3 Hubei Hongshan Laboratory, Wuhan 430070, China;; 4 Department of Entomology and Nematology, University of California, Davis, California 95616, USA;; 5 Institute of Weed Science, Entomology and Plant Pathology, University of the Philippines Los Baños, Laguna 4031, Philippines;; 6 Wuhan Academy of Agricultural Sciences, Wuhan 430065, Hubei, China

## Abstract

The remarkable ability to survive desiccation and persist in a dry state is among nature's most fascinating adaptations, enabling certain organisms to withstand extreme dehydration without damage. This phenomenon has been widespread across diverse life forms, including plants, fungi, and nematodes. However, our understanding of its molecular basis, particularly in animals, remains limited. *Aphelenchus avenae* nematodes are notable for their exceptional tolerance to dehydration, and multiple genes related to this trait have been identified. However, the absence of a chromosome-scale, high-contiguity genome for *A. avenae* has been a limitation in the genome-wide identification of gene families potentially involved in desiccation tolerance. In this study, we assemble a high-quality, telomere-to-telomere haplotype genome of *A. avenae*. Transcriptomic analyses reveal distinct sets of genes involved in responses to desiccation and freezing stress. Notably, under desiccation stress, several desiccation-tolerance genes exhibit allelic imbalance expression. Among these, we identify the stress response gene *P5CS* in *A. avenae* nematodes. *Aap5cs* RNAi experiments demonstrate that knockdown of *Aap5cs* results in increased accumulation of reactive oxygen species under desiccation stress and reduced desiccation survival time, suggesting that *Aap5cs* plays a role in the stress response of *A. avenae*. This finding also raises the possibility of functional parallels between plant and nematode responses to dehydration. This study enhances our understanding of nematode resistance to desiccation stress and provides valuable genetic resources for investigating the intricate regulatory pathway that organisms use for dehydration stress adaptation.

Adaptation to water scarcity or extreme environments is widely observed across diverse organisms. Many plants, insects, invertebrates, and even microbes exhibit notable drought tolerance (Alpert 2006). Most species survive drought by utilizing strategies that prevent internal water loss. There is only a smaller subset of species that can endure complete desiccation and be revived thereafter. This rare capability is known as desiccation tolerance or anhydrobiosis (Alpert 2005). Many of these organisms are only desiccation tolerant during their early life stages, such as seeds (de Almeida Garcia Rodrigues et al. 2022) and eggs (Prasad et al. 2023). However, three notable invertebrates are known to tolerate desiccation at any stage of their life cycle including adulthood. These are bdelloid rotifers (Pouchkina-Stantcheva et al. 2007; Simion et al. 2021), nematodes (Browne et al. 2002; Wan et al. 2021), and tardigrades (Lim et al. 2024).

In recent years, the mechanisms of desiccation tolerance in organisms have been extensively studied, including the elaborate coordinated regulation of multiple complex components. These components are comprised of certain proteins (Chakrabortee et al. 2007, 2010; Hand et al. 2011), cellular osmotic regulators (Crowe et al. 2001), and antioxidant enzymes (Xing et al. 2024). The Late Embryogenesis Abundant (LEA) protein was the first protective protein described in desiccated plant seeds (Baker et al. 1988) and was subsequently confirmed to play a role in desiccation tolerance in animals such as nematodes (Browne et al. 2002). The LEA proteins eliminate the aggregation of water-soluble proteins in cells caused by desiccation which increases cytoplasmic viscosity. It also participates in repairing desiccation-induced DNA damage that occurs during dehydration (Chakrabortee et al. 2007, 2010; Belott et al. 2020; Abe et al. 2024). In other organisms, desiccation tolerance is induced by the replacement of water in the body with glassy sugars. One of the important sugars that these organisms utilize to resist desiccation is trehalose (Hespeels et al. 2015; Chen et al. 2018). Trehalose provides the main energy source in the early stages of hydration and helps restore their antioxidant potential through the pentose phosphate pathway (Ryabova et al. 2020). Osmotic stress is another major challenge in desiccation tolerance. To resist osmotic stress, plants accumulate proline (Burton 1991). The proline biosynthetic enzyme known as delta-1-pyrroline-5-carboxylate synthetase (P5CS) plays a crucial role in plant responses to salt and drought stress aside from mitigating oxidative stress (Székely et al. 2008). Although numerous mechanisms of desiccation tolerance have been identified across diverse life forms, including plants, animals, and microorganisms, these mechanisms appear to share common features and rely on highly coordinated genetic regulatory interactions. To date, a comprehensive framework that fully explains these interactions is lacking, leaving substantial room for further investigation in this field.

Nematodes are one of the largest phyla of animals, with millions of species. So far, many nematodes have been reported to have the ability to tolerate desiccation. These include *Panagrolaimus* species (Shannon et al. 2008), *Oscheius tipulae* (Torrini et al. 2016), *Plectus murrayi* (Adhikari et al. 2010), *Aphelenchoides besseyi* (Chen et al. 2018), *Ditylenchus destructor* (Ma et al. 2020), *Bursaphelenchus xylophilus* (Hoover et al. 2010), and *Aphelenchus avenae* (Browne et al. 2002; Chakrabortee et al. 2010; Banton and Tunnacliffe 2012; Wan et al. 2021). Among them, *A. avenae* is the most studied desiccation-resistant nematode. The first draft genome of *A. avenae* was reported by Wan et al. (2021). In their study, gene family expansions and transcriptomic dynamics during anhydrobiosis were highlighted, and species-specific intrinsically disordered proteins associated with desiccation tolerance were identified. In addition, several molecular components including LEA proteins, trehalose-6-phosphate synthase (TPS), mitogen-activated protein kinase (MAPK), and glycosyltransferases have been reported to participate in desiccation resistance (Browne et al. 2002; Goyal et al. 2005; Banton and Tunnacliffe 2012). However, the absence of high-quality, chromosome-level genome assemblies has thus far restricted more detailed investigations into the underlying regulatory mechanisms. In recent years, more haplotype genomes have been analyzed which provide valuable insights into domestication (Li et al. 2024c; Su et al. 2024), hybridization (Dai et al. 2023; Li et al. 2024a), chromatin architecture (Li et al. 2024b; Lin et al. 2024), evolution, and freezing tolerance (Dong et al. 2024; Feng et al. 2024a; Tan et al. 2024). To comprehensively elucidate the molecular mechanisms underlying desiccation tolerance in nematodes, this study aims to assemble haplotype-resolved genome of *A. avenae*, generate transcriptomic data sets across developmental stages, and analyze allelic expression preferences under desiccation stress. By integrating knowledge of desiccation-related genes from plants and animals, we systematically identify candidate genes in the haplotype genome and functionally validate key regulators of desiccation tolerance. This will provide a theoretical framework for understanding the molecular basis of desiccation tolerance in animals.

## Results

### Haplotype-resolved 3D genome construction and annotation

**Figure 1.**
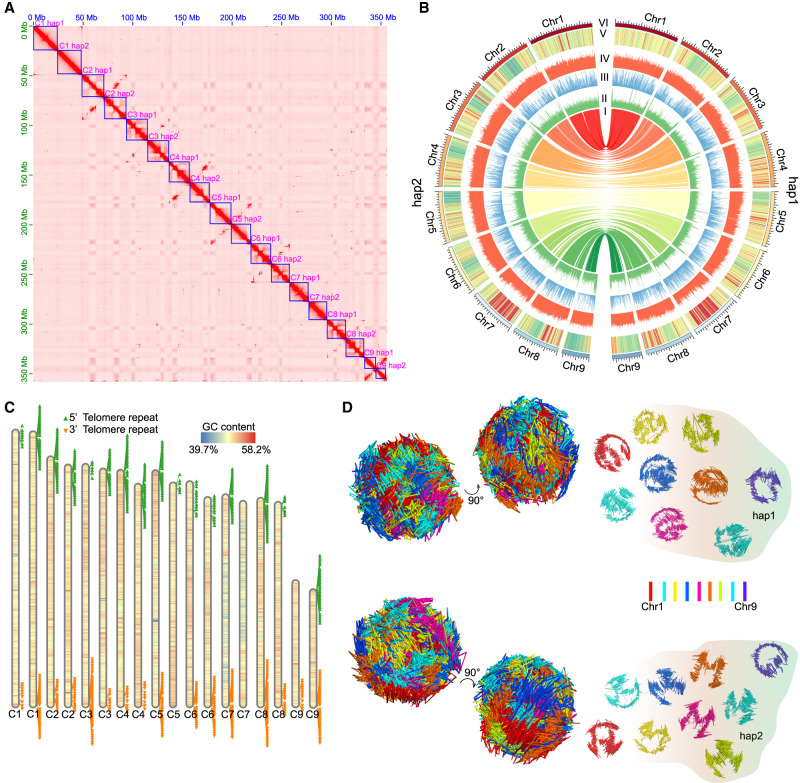
Construction of the haplotype-reserved 3D genome architecture of *A. avenae*. (*A*) Hi-C heat map of two haplotype sets of *A. avena*e; from *top* to *bottom* are nine pairs of homologous chromosomes 1–9. (*B*) Circos plot (Krzywinski et al. 2009) of *A. avenae* haplotype-resolved T2T genomic features. I: The collinearity between two sets of haplotypes; II: Distribution and abundance of DNA 6mA in chromosomes; III: Distribution and abundance of TE in chromosomes; IV: Distribution and abundance of GC content in chromosomes; V: Heat map of gene expression in a 50-kb window across the genome; VI: The outermost track exhibits the chromosome karyotype in units of 1 Mb. (*C*) The relative lengths of chromosomes and the distribution of telomeres of the two haplotypes. Each triangle represents two telomeric repeat sequences. The adjacent chromosomes are designated hap1 and hap2, respectively. (*D*) 3D genome structure of two haplotypes at the mix stage of *A. avenae*. (*Left*) Whole genome; (*right*) the nine autosome pairs visualized separately.

Based on phylogenetic analysis of 28S rRNA sequences, the nematode used in this study was confirmed to be *Aphelenchus avenae* (Supplemental Fig. S1). We generated 35 GB Pacific Biosciences (PacBio) data, 20 GB Oxford Nanopore Technologies (ONT) data, 75 GB Hi-C data, and 16 GB Illumina short-reads data to assemble the *A. avenae* genome. We assessed the genome ploidy of *A. avenae* and confirmed it to be diploid (Supplemental Fig. S2A). The haploid genome size was estimated to be ∼173 Mb (Supplemental Fig. S2B). Furthermore, *k*-mer–based analyses revealed a high level of genomic heterozygosity (∼6%) (Supplemental Fig. S2B), which increased the likelihood of successfully assembling its haplotype-resolved genome. We first used Flye to assemble the PacBio data and obtained a 350-Mb genome with an N50 of 1.1 Mb (*n* = 89), with a total number of contigs of 1048. To improve the quality of the draft assembly, we reassembled the genome with ONT data and enhanced the genome using the PacBio assembly coupled with Illumina data-based correction. The assembly size was 356 Mb with a contig N50 of 2.7 Mb (*n* = 38), which is 19 times higher than the previous assembly with an N50 of 142 kb (*n* = 385) (Wan et al. 2021). Based on the Hi-C data, we successfully anchored the 99.5% of sequence to 18 chromosomes (nine pairs of homologous chromosomes) (Fig. 1A). Because the two sets of homologous chromosomes could not be distinguished, we designated the slightly larger chromosome as haplotype 1 (hap1) and the slightly smaller one as haplotype 2 (hap2) within each homologous pair (Fig. 1A). Finally, the assembled genome size of hap1 and hap2 were 181,012,927 bp and 174,343,290 bp, respectively. Analysis of read depth revealed that the depth across individual chromosomes matched the overall genome depth (Supplemental Fig. S3). Our final assembly showed a high completeness assessment by BUSCO value with 80.2% for the whole genome, 72.3% for hap1, and 70.5% for hap2 (Supplemental Fig. S4), which was higher than the published *A. avenae* (Wan et al. 2021) and root-knot nematodes (Dai et al. 2023).

By integrating multiple transcriptome data sets, we annotated 58,330 genes in the *A. avenae* genome, which is ∼35% more than reported in the previous version, with 29,477 genes assigned to hap1 and 28,547 to hap2. Clustering analysis of gene expression levels across different stages revealed distinct clusters of highly expressed genes specific to each stage (Supplemental Fig. S5). GO enrichment analysis of stage-specific genes revealed distinct biological signatures across development, with J2-expressed genes enriched for behavior, J3/J4 genes enriched for development, and female- and male-expressed genes enriched for reproductive functions (Supplemental Fig. S6). In addition, genes highly expressed under drought/cold stress were mainly enriched in response to antioxidant activity, superoxide, response to oxidative stress, and defense response (Supplemental Fig. S6).

Collinearity analysis showed that homologous chromosomes had strong collinearity (Fig. 1B; Supplemental Fig. S7A,B). We identified 20,288 orthologous gene pairs between the two haplotypes, whereas 8550 genes were unique to hap1, and 8203 genes were unique to hap2. GO enrichment analysis on the genes present only in hap1 or hap2 also revealed that the hap1-specific genes were predominantly enriched in biological processes related to nucleic acid and protein binding (Supplemental Fig. S8A), whereas the hap2-specific genes were primarily enriched in kinase binding and various enzymatic activities (Supplemental Fig. S8B). These functional differences therefore illustrate haplotype-level gene content asymmetry arising from the high degree of heterozygosity and structural variation, rather than true subgenome specialization. Subsequently, transposable elements (TEs) were annotated, with 133,375 and 127,491 elements identified in hap1 and hap2, respectively. The distribution of different types of TEs between chromosomes shows slight variations (Supplemental Fig. S9).

To evaluate the completeness of the assembled genomes, repetitive sequences were surveyed across each chromosome, revealing that most chromosomes contained the typical telomeric repeat motif (TTAGGC) at both ends (Fig. 1C; Wicky et al. 1996). We also noticed that, in several pairs of homologous chromosomes, only one of the two copies assembled had a relatively complete telomere sequence. This likely occurred because repetitive regions, such as telomeres, pose well-known challenges for phasing during genome assembly. In the Hi-C anchoring step, most telomere sequences generate signals on both chromosomes simultaneously, yet they are ultimately assigned to only one chromosome (Supplemental Fig. S10). To further explore the genomic landscape of *A. avenae*, we performed a Pfam search and annotation on all its protein sequences and discovered five potential DNA 6mA demethylases with the 2OG_FeII_oxy2 motif, as we previously described (Dai et al. 2026). Using PacBio Sequel I data, we were able to identify 6mA sites with individual base pair resolution. After filtering out low-quality 6mA sites, we generated 197,985 reliable 6mA sites. Here, we found that most chromosomes had relatively high 6mA levels at their ends (Fig. 1B), which may be related to the function of telomeres.

Next, we reconstructed the 3D genome architecture for each haplotype chromosome at 10-kb intrachromosomal and 100-kb interchromosomal resolution (Fig. 1D). In each haplotype, some chromosomes exhibited similar 3D structures. For example, Chr 1, Chr 5, and Chr 6 in hap1 were similar, whereas Chr 1, Chr 5, Chr 6, and Chr 7 in hap2 shared similarities. We observed that the 3D structure between homologous chromosomes was not conserved, with only Chr 7 maintaining a similar 3D structure (Fig. 1D). To evaluate whether this pattern was influenced by haplotype phasing, we compared 3D structures reconstructed from each haplotype independently with those derived from joint prediction and found the results to be identical. We then hard-masked all regions that could not be aligned between homologous chromosomes. Following hard-masking, the jointly inferred 3D structures changed substantially for most chromosomes, and the structural similarity between homologous chromosome pairs increased markedly, particularly for Chr 1, Chr 2, Chr 5, Chr 6, and Chr 9 (Supplemental Fig. S11).

### Evolutionary analysis between haplotype genomes

**Figure 2.**
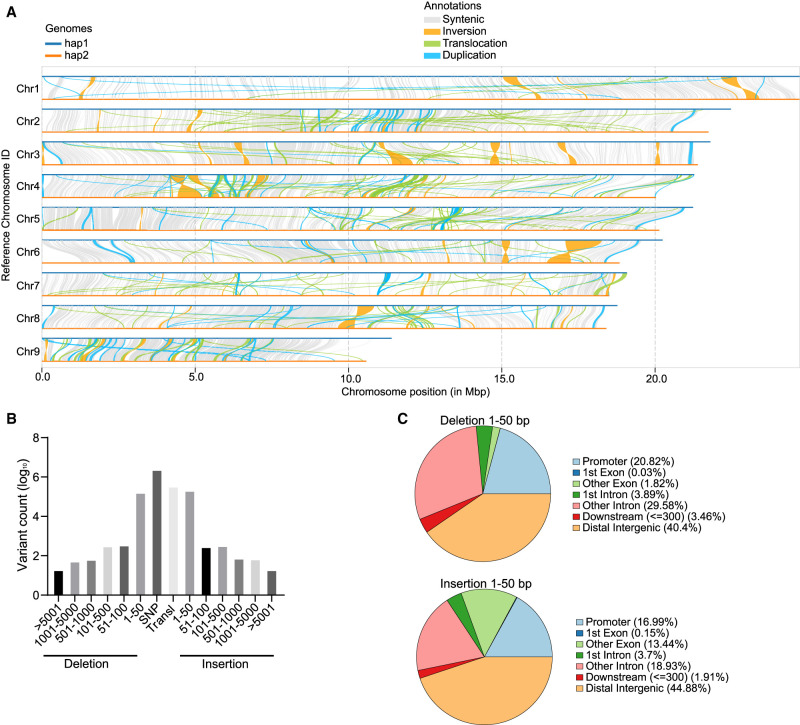
Structural variations between haplotypes. (*A*) Syntenic analysis of the *A. avenae* genome. (*B*) The distribution of variation length was shown using the hap1 genome as reference, and ordinate numbers were logarithmically transformed. (*C*) Genome-wide annotation of deletion and insertion mutations smaller than 50 bp.

To accurately assess the divergence between the genomes of the two haplotypes, we analyzed polymorphisms across the nine pairs of homologous chromosomes. The results showed that there were many structural variations between each pair of homologous chromosomes (Fig. 2A). We subsequently evaluated the average nucleotide identity (ANI) between chromosomes and homolog gene pairs. The obtained ANI between chromosomes was relatively low, ranging from 85.98% to 93.41% (Supplemental Fig. S12A), whereas the ANI between homologs ranged from 91.7% to 96.4% (Supplemental Fig. S12B,C). These results suggest that *A. avenae* may have originated from interspecific hybridization. It has been reported that *A. avenae* has two populations: sexual and parthenogenetic (Fisher and Triantaphyllou 1976). Therefore, the sequence divergence observed may also be due to long-term parthenogenesis which can cause homologous chromosomes to accumulate independent mutations and diverge substantially due to lack of recombination (Mark Welch and Meselson 2000). Additionally, a comprehensive analysis revealed 2,330,360 SNPs, 204,030 insertions (1–50 bp), 162,675 deletions (1–50 bp), 1547 structural variants (SVs, >50 bp), 444,289 inversions, and 334,833 translocations within the syntenic blocks of the assemblies (Fig. 2B). We observed that most insertions and deletions were predominantly under 50 bp in length, with most mutation sites located in intergenic regions (Fig. 2C). Because insertions and deletions in exon regions will directly affect the encoded amino acid sequence of the gene, we performed GO enrichment analysis on the functions of these genes. The results showed that these genes were enriched in several important functions such as chromosome organization, nuclear migration, and glucosidase activity (Supplemental Table S1).

We subsequently analyzed the expression and functional differences between alleles within the haplotypes. Expression levels of alleles were measured across each developmental stage and condition, allowing us to identify differentially expressed alleles (DEAs). Specifically, we detected 2642, 3004, 4329, 5396, 4924, 4713, and 3615 DEAs in the J2, J3/J4, male, female, mixed stage, drought stress, and 4°C low-temperature stress conditions, respectively. Among the DEAs across different developmental stages, 907 genes were shared across all stages, whereas the mixed stage and female stage showed the highest numbers of unique genes, with 1567 and 1218 unique DEAs, respectively (Supplemental Fig. S13A). The GO enrichment analysis of the 907 genes common to each developmental stage revealed that these genes are associated with regulation of development and growth, behavior, and reproductive processes (Supplemental Table S2). Additionally, 2339 DEAs were shared between drought and cold stress conditions. Compared to DEAs under normal conditions (mixed stage), there were 1502 and 1023 unique DEAs specific to drought and cold stress, respectively (Supplemental Fig. S13B). GO functional analysis indicated that drought-specific DEAs included many genes involved in drought resistance, such as UDP-glycosyltransferase activity (Feng et al. 2024b) and regulation of stress responses (Supplemental Fig. S13C).

To evaluate the natural selection of alleles, *K*_a_ and *K*_s_ values were calculated for all allelic pairs. Among these alleles, only 1.03% (194 out of 18,796) showed evidence of positive selection (*K*_a_/*K*_s_ > 1). We compared the differences in *K*_a_/*K*_s_ values between DEAs and equivalently expressed alleles (EEAs) in all stages and found that DEAs showed higher *K*_s_ and *K*_a_/*K*_s_ values than EEAs (Supplemental Fig. S14). These results suggest that the DEAs may have experienced positive selection during evolution and may play an important role in the growth and development of nematodes and their resistance to adverse environments. In DEAs and EEAs, the proportion of genes showing evidence of positive selection (*K*_a_/*K*_s_ > 1) was 0.97% and 0.95%, respectively, whereas the proportion that underwent clear purification selection (*K*_a_/*K*_s_ < 0.1) was 22.1% and 38.1%, respectively. The rate of genes showing evidence of positive selection was similarly low in both groups, indicating that DEAs and EEAs may be subject to relatively weak selection pressure during adaptive evolution. However, EEAs seem to experience stronger purifying selection, which suggests that these genes must maintain functional stability throughout evolution, possibly due to their greater importance in biological processes, making them less tolerant of mutations.

To further investigate the evolutionary relationships of *A. avenae*, we performed chromosome-level synteny comparisons between *A. avenae* and several other migratory plant-parasitic nematode species. First, we examined the synteny between *A. avenae* and two evolutionarily earlier species, *B. xylophilus* and *A. besseyi* (Qing et al. 2025). The results revealed limited syntenic blocks between *A. avenae* and these two species with only five blocks being shared among all three nematodes (Supplemental Fig. S15A). This suggests that they may have originated from distinct evolutionary lineages. We then compared *A. avenae* with the relatively closely related species *D. destructor* (Chen et al. 2025) and found extensive synteny across most chromosomes, except for Chromosome 1 of *A. avenae*, which showed little to no collinearity with *D. destructor* (Supplemental Fig. S15B). Furthermore, analysis of Nigon elements (Gonzalez de la Rosa et al. 2021) in *A. avenae* indicated that Chromosomes 1 and 7 contain only a small number of Nigon elements, whereas other chromosomes harbor large regions with low Nigon element density (Supplemental Fig. S16). These observations suggest that parts of the genome may have a complex evolutionary origin, potentially involving hybridization or contributions from lineages with reduced or divergent Nigon element content, although further studies are required to clarify their precise ancestry. Notably, Nigon X, which marks the X Chromosome (Gonzalez de la Rosa et al. 2021), was identified only on Chromosome 2 of *A. avenae*. Together with the synteny analysis between *A. avenae* and *D. destructor*, we infer that Chromosome 2 of *A. avenae* represents its X Chromosome (Supplemental Fig. S15B).

### Secreted proteins evolve rapidly to increase fitness of *A. avenae*

The analysis of high-confidence homologous genes revealed that the majority (7392 gene pairs, 46.6%) have identical amino acid lengths but differ in sequence. A considerable proportion (29.4%) corresponded to allelic genes in which one haplotype exhibits complete coverage, whereas the other shows partial coverage. In contrast, fully identical homologous gene pairs—those with both identical lengths and amino acid sequences—accounted for only 6.5% (1027 pairs). These results suggest that there is a certain degree of conservation between homologous genes. However, with evolution and genomic changes, some gene pairs will have sequence differences or asymmetry between homologous chromosomes. This variation may be related to the adaptive evolution of different species or the functional differentiation of specific genes.

**Figure 3.**
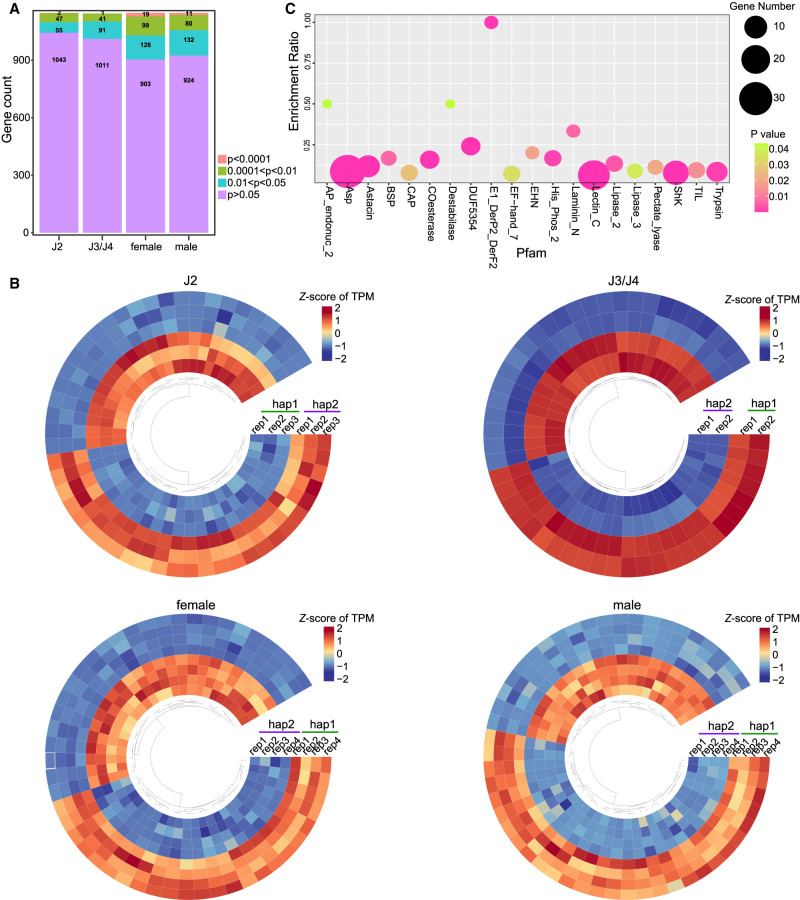
Allelic imbalance in expression of secreted proteins. (*A*) Statistics of the number of allele expression differences among secreted protein with the same length but sequence variation at four ages: J2, J3/J4, female, and male. The significance of expression differences between replicates at different stages was calculated by *t*-test, and *P* < 0.05 was considered to be under allelic expression imbalance. (*B*) The top 50 secretory proteins with allelic imbalance expression at each developmental stage were used for heat map display, and no gene expression bias towards a certain haplotype was found. (*C*) Pfam domain analysis of secretory proteins with allelic imbalance expression revealed that they were mainly enriched in nematode feeding-related functional genes.

For alleles with completely identical sequences, we speculated that they were mainly functionally conserved—in other words, housekeeping genes. Therefore, we performed Pfam domain enrichment analysis on the above 1027 alleles with completely identical sequences. The results showed that these genes were mainly enriched in ribosome, ATP synthesis, tubulin, sperm motility, centromere, and pre-mRNA splicing (Supplemental Fig. S17A). This differed from the Pfam functional enrichment for the alleles with coverage <80% (Supplemental Fig. S17B). Considering that genes with faster evolutionary rates may have undergone more mutations, we also conducted a prediction analysis for secreted proteins in *A. avenae*. We identified 3509 (hap1) and 3411 (hap2) secreted proteins that are evenly distributed across chromosomes (Supplemental Fig. S18). Further analysis revealed that only 56 (1.6%) of the secreted proteins had identical sequences with their alleles, whereas 1147 secreted proteins had identical lengths but sequence variations. We speculate that some of these secreted proteins with sequence divergence may have undergone functional and/or expression differentiation. To explore this, we conducted an analysis of expression differences for these 1147 alleles. We observed that some genes exhibited allelic imbalance in expression (Fig. 3A). However, this imbalance did not consistently favor one haplotype over the other across developmental stages (Fig. 3B), with the numbers of hap1-and hap2-dominant genes being broadly comparable. Specifically, in the J2 stage, hap2-biased genes are slightly more abundant, whereas in males, hap1-biased genes show a modest increase. These opposing patterns indicate that allelic imbalance varies by life stage rather than consistently favoring one haplotype. Because haplotype designations were assigned arbitrarily, these patterns do not support a systematic association between allelic dominance and a specific haplotype set. Pfam analysis showed that these imbalanced expression secretory proteins were enriched in aspartyl protease (Asp), trypsin, digestive enzyme (Astacin), esterase (COesterase), pectate lyase, xylose isomerase (AP_endonuc_2), lectin, and other functions (Fig. 3C). All these genes are important for the fitness of *A. avenae*.

### Desiccation-tolerant alleles exhibit imbalanced expression under drought stress

**Figure 4.**
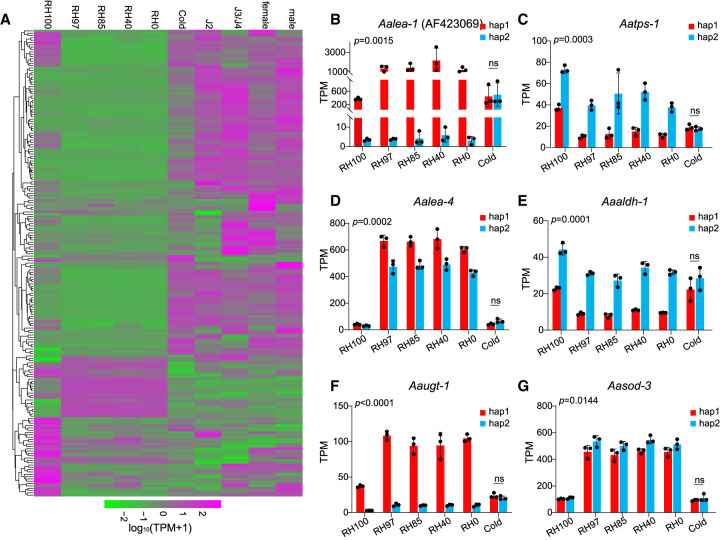
Expression landscape and allelic imbalance expression of desiccation tolerance–related genes under desiccation stress. (*A*) Clustering of expression profiles of genes that may be related to nematode resistance to desiccation stress at different developmental stages and under different desiccation stressors showed that these genes had significantly different expression patterns under desiccation stress, normal growth, and cold stress. (*B*–*G*) The alleles of genes associated with desiccation stress response were expressed in an unbalanced manner under different desiccation stress conditions, whereas these genes did not exhibit such unbalanced expression under cold stress. Nematodes may use specific gene regulatory mechanisms to enhance fitness. Bars represent mean values ± SD. *P* values for the entire data set were calculated using two-way ANOVA, whereas *P* values for the cold condition were calculated using a two-tailed unpaired Student's *t*-test. (ns) no significant difference.

To analyze the gene expression regulation of *A. avenae* under drought stress, we first surveyed the literature on desiccation-related genes reported in nematodes, plants, rotifers, and tardigrades (Browne et al. 2002; Pouchkina-Stantcheva et al. 2007; Székely et al. 2008; Ryabova et al. 2020; Wan et al. 2021; Lim et al. 2024). Based on these studies, we focused on representative genes from several stress-related gene families, including *lea-1* (late embryogenesis abundant protein), *sod-3* (superoxide dismutase), *aldh-1* (aldehyde dehydrogenase), *tps-1* (trehalose-6-phosphate synthase), and *ugt-1* (UDP-glycosyltransferase). Homologous genes were then identified in the *A. avenae* genome by sequence comparison, and conserved domains of reported desiccation-related genes were summarized to further screen the Pfam-based genome-wide protein annotation. Genes containing these conserved domains were extracted and merged with the homolog-based candidates, resulting in a final set of 204 potential desiccation-related genes. We performed a clustering analysis of the expression levels of these 204 genes across different developmental stages and under varying humidity conditions. The results indicated that genes highly expressed under drought stress showed low expression across developmental stages under normal conditions and vice versa (Fig. 4A). Moreover, the results showed that most genes were downregulated under drought stress, and only 13.7% (28 out of 204) genes were upregulated in this process (Fig. 4A). Further studies have demonstrated that genes from the five above-mentioned families were significantly upregulated under drought stress (Supplemental Fig. S19A–F). Additionally, there was a notable allelic imbalance in their expression during drought stress, but no such imbalance was observed under cold stress conditions (Fig. 4B–G; Supplemental Fig. S19A–F). The first desiccation tolerance gene *lea-1* (Browne et al. 2002) in nematodes reported by previous researchers showed extremely significant upregulation under drought stress, and the expression difference between alleles was as high as hundreds of times (Fig. 4B; Supplemental Fig. S19A). Additionally, the *lea-1* gene exhibited higher expression of the hap2 allele during normal growth in nematodes, whereas the hap1 allele showed higher expression under drought stress (Supplemental Fig. S19A). However, only a small portion of alleles exhibit imbalanced expression during normal growth. For instance, another *lea-4* gene identified in this study, as well as *sod-3*, showed balanced expression across developmental stages and under 100% relative humidity (RH100) conditions. Significant upregulation and allele-specific expression imbalance was observed as RH levels continued to decrease (Supplemental Fig. S19C,F). These results suggest that allelic differences in gene expression may play a role in the response of *A. avenae* to drought stress.

### *Aap5cs* gene is involved in desiccation tolerance

**Figure 5.**
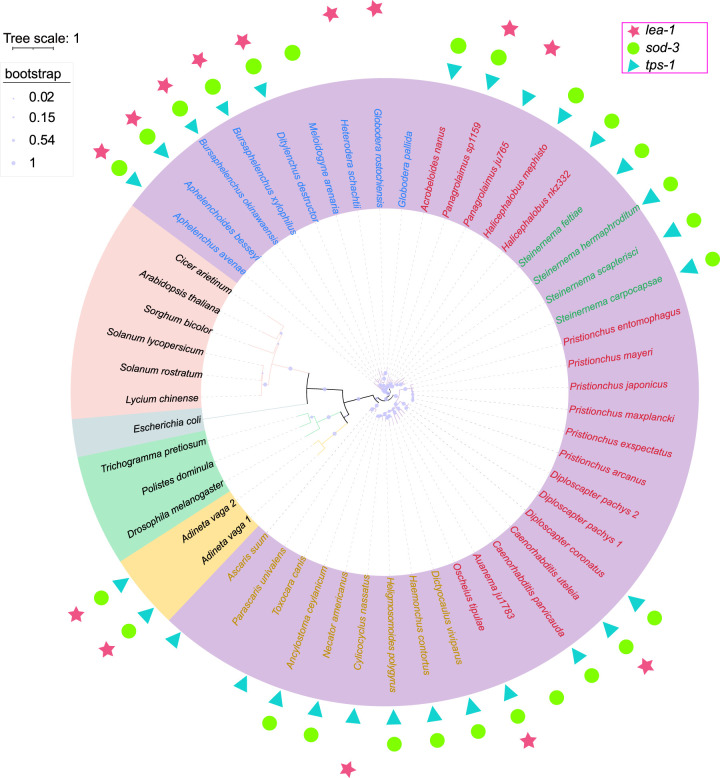
Phylogenetic tree of *P5CS* genes in nematodes, insects, and plants. The *P5CS* genes of plants, insects, and rotifers were downloaded from NCBI Gene. The *P5CS* gene presence in nematodes was confirmed using *Aap5cs* as query, and protein alignment by WormBase ParaSite blast to identify the homologous sequences in other nematodes. The presence of *lea-1*, *tps-1*, and *sod-3* genes in other nematodes was determined by using *A. avenae* and *Caenorhabditis elegans* genes as queries in WormBase ParaSite blast. *E. coli* glutamate-5-semialdehyde dehydrogenase (PorA) was used as an outgroup. Different background colors represent different species, including pink for plants, green for insects, purple for nematodes, yellow for rotifers, and gray for *E. coli*. Among nematodes, blue fonts represent plant-parasitic nematodes, red fonts represent free-living nematodes, green fonts represent entomopathogenic nematode, and yellow fonts represent animal-parasitic nematodes.

During our analysis of potential drought tolerance genes, we discovered a gene that was implicated in desiccation tolerance in plants, *DELTA1-PYRROLINE-5-CARBOXYLATE SYNTHETASE* (*P5CS*) (Székely et al. 2008), which has not been studied in nematodes. This *P5CS* gene in *A. avenae* (*Aap5cs*) contains both an aldehyde dehydrogenase domain and a proline synthase domain, and only one copy in hap1, and no copy in hap2. We blasted the *Aap5cs* gene in WormBase ParaSite and found that 37 species of nematodes have this gene, including 17 free-living nematodes, nine animal-parasitic nematodes, eight plant-parasitic nematodes, and four insect-parasitic nematodes. Although there are reports that the *P5CS* gene originated from horizontal gene transfer (HGT) (Filgueiras et al. 2024), our predicted results of HGT genes for *A. avenae* did not include the *Aap5cs* gene. Nevertheless, we sought to investigate its relationship to homologous genes in plants, insects, nematodes, and rotifers. We downloaded homologous genes from selected plants and insects and used *Escherichia coli* glutamate-5-semialdehyde dehydrogenase (PorA) as outgroups to construct a phylogenetic tree of *P5CS* (Fig. 5; Supplemental Fig. S20). The phylogenetic tree of *P5CS* across species indicates that the *P5CS* genes are conserved across diverse lineages but may have undergone lineage-specific divergence after arising from a common ancestral gene (Fichman et al. 2015). Within nematodes, the phylogenetic tree can be divided into four major branches. Plant-parasitic nematodes cluster together with four free-living nematodes that are evolutionarily close to them, forming one branch. Insect-parasitic nematodes constitute a separate branch. Most free-living nematodes, together with animal-parasitic nematodes, form another branch, and three animal-parasitic nematodes group into an independent branch (Fig. 5). This suggests that *P5CS* may have undergone multiple independent differentiation events during its evolutionary history.

**Figure 6.**
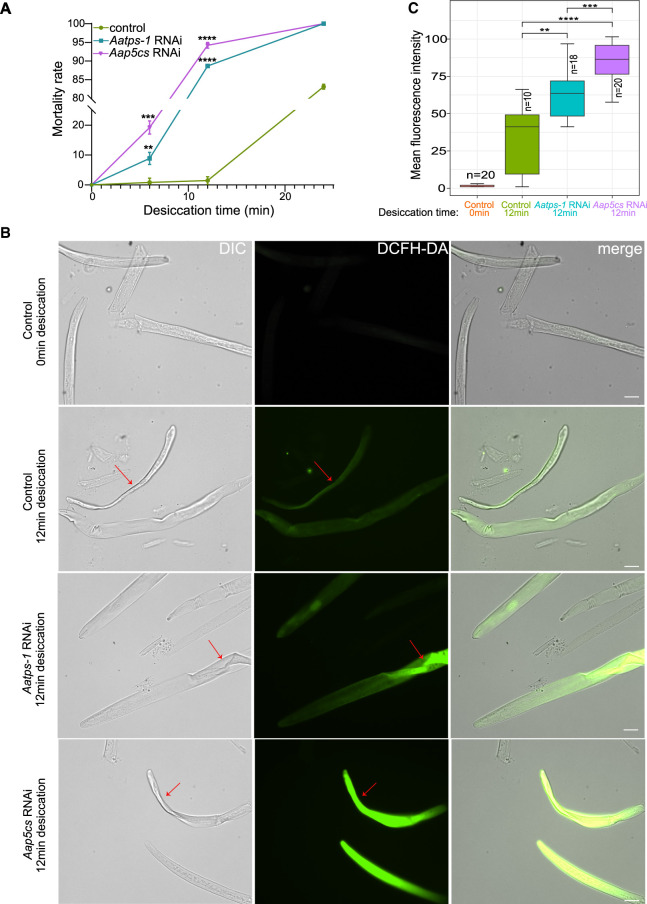
*Aap5cs* and *Aatps-1* contribute to the desiccation-tolerance in *A. avenae*. (*A*) Mortality rates of nematodes subjected to different desiccation times after RNAi targeting *Aap5cs* and *Aatps-1*. The ds*gfp* RNA was used as a control. The significance analysis was performed between the treatment group and the control group using a *t*-test. (**) *P* < 0.01, (***) *P* < 0.001, (****) *P* < 0.0001. (*B*) After drying for 12 min, the nematodes with different treatments were incubated with the DCFH-DA probe to detect the level of ROS in the nematode's body. The red arrow indicates the dehydrated regions on the nematode. Bar = 20 µm. (*C*) Quantification of pixel intensity of ROS signal in *A. avenae* body. *P*-values were calculated by a two-sided Wilcoxon rank-sum test.

To verify whether *Aap5cs* is involved in the nematode *A. avenae*’s response to desiccation stress, we performed RNAi-mediated gene silencing and then examined the phenotypes of nematodes under desiccation stress. We used another gene, *Aatps-1*, previously reported to be associated with desiccation resistance in *A. avenae* (Goyal et al. 2005), as a positive control. The results showed that nematodes directly exposed to 40% relative humidity for 12 min could recover upon rehydration (Fig. 6A). However, after RNAi targeting *Aap5cs* or *Aatps-1*, their recovery was significantly reduced (Fig. 6A; Supplemental Fig. S21). Furthermore, we measured the levels of reactive oxygen species (ROS) in *A. avenae* under different treatment conditions using the DCFH-DA method to assess their ability to mitigate ROS accumulation during desiccation stress. For the control group of nematodes without RNAi treatment, the results showed no detectable ROS signals in nematodes subjected to 0 min of desiccation treatment, and a slight accumulation of ROS was observed after 12 min of desiccation (Fig. 6B). However, when *Aatps-1* or *Aap5cs* was silenced, strong ROS accumulation was detected in *A. avenae* both after 6 and 12 min of desiccation, particularly in the nematode trunks exhibiting evident dehydration where extensive ROS signals were observed (Fig. 6B; Supplemental Figs. S22–S24). Statistical analysis revealed that silencing *Aap5cs* and *Aatps-1* resulted in significantly higher ROS accumulation in nematodes under desiccation stress compared to the control group (Fig. 6C). Moreover, ROS levels in the *Aap5cs* RNAi treatment group were significantly higher than those in the *Aatps-1* RNAi group (Fig. 6C), suggesting that *Aap5cs* may play a more critical role than *Aatps-1* in *A. avenae* responses to desiccation induced ROS accumulation.

## Discussion

The desiccation tolerance mechanism of nematodes has become a primary focus among biologists. Their ability to survive for a long time in extreme drought conditions has also become an important reference value for the drought response of ecosystems (Hand et al. 2011). In the event of desiccation, organisms face multiple challenges, including cell dehydration, oxidative stress, ROS accumulation, DNA damage, osmotic pressure imbalance, and the need to maintain cell membrane stability and detoxify harmful metabolites (Hand et al. 2011). Although many studies have identified genes and pathways associated with desiccation tolerance (Browne et al. 2002; Alpert 2005, 2006; Goyal et al. 2005; Chakrabortee et al. 2010; Ryabova et al. 2020; Lim et al. 2024), they cannot fully explain this phenomenon. Plants naturally possess more structural and biochemical advantages in resisting drought (Hoekstra et al. 2001), such as hard seed coats, waxy cuticles, and polysaccharide-rich cell walls. In contrast, animals lack these protective barriers, making their molecular mechanisms of desiccation tolerance particularly intriguing and a potential source of inspiration for biomimetic strategies in agriculture and biotechnology.

In this study, we uncovered allelic imbalance in the expression of desiccation tolerance genes in *A. avenae* under stress and identified a range of genes potentially involved in this process. Importantly, we demonstrated that the *P5CS* gene widely documented in plants also plays a pivotal role in nematode desiccation tolerance. These findings provide new insights into the mechanisms underlying this trait in animals and lay the foundation for further exploration. *P5CS* has been linked to ROS regulation in plants, as proline can enhance catalase activity (Bauduin et al. 2022). In *Meloidogyne incognita*, catalase can be secreted into the host to modulate ROS levels (Zhu et al. 2024). In contrast, in *A. avenae*, catalase homologs show a gradual decrease in expression under drought stress, and these catalases lack a secretion signal peptide, indicating that they function intracellularly in ROS detoxification. Together, these results indicate that *Aap5cs* may contribute to maintaining osmotic balance and enhancing catalase activity to mitigate oxidative stress during desiccation. However, the precise mechanism of ROS accumulation after *Aap5cs* knockdown requires further investigation.

Beyond individual genes, an important unresolved question is how these desiccation-related genes are coordinately regulated. Desiccation tolerance in organisms is a finely tuned and complex process (Potts 2001). Whether specific desiccation sensors activate transcription factors and signaling cascades, and how allelic expression bias contributes to this regulation, remains to be discovered. Although we detected allele-specific expression under stress, the random division of homologous chromosomes in our data set prevents us from determining whether dominant expression consistently favors one haplotype. Future studies should employ haplotype-resolved genomes in desiccation-tolerant species to test whether biased allele expression is a universal feature of this process.

Our findings suggest that organisms with a greater repertoire of desiccation-related genes exhibit stronger drought resistance. For instance, several desiccation-tolerant organisms have been reported to exhibit coordinated upregulation or stress-responsive expression of genes. Combined with the results of Figure 5, we speculate that *Bursaphelenchus okinawaensis* and *Caenorhabditis uteleia* could also tolerate desiccation. However, determining which of these genes are necessary or sufficient for survival will require future investigation.

The evolution of desiccation tolerance is closely linked to ecological adaptation, and nematodes serve as a powerful model system for investigating these processes. By employing desiccation-associated genes as molecular markers in combination with life history traits and population genetic analyses, it may be possible to identify species or populations exhibiting enhanced tolerance and to elucidate the underlying genetic architecture. Ultimately, comprehensive dissection of the genes and regulatory pathways governing desiccation tolerance holds promise for informing biomimetic strategies and guiding genetic interventions aimed at improving drought resilience in agricultural and biotechnological applications.

## Methods

### Nematode culture and collection

*A. avenae* nematodes were propagated in the laboratory in *Botrytis cinerea* cultures on potato dextrose agar (PDA). Briefly, a small piece of about 0.5 cm square was cut from *Botrytis cinerea* pure culture and then transferred to fresh PDA medium. After culturing it in the dark at 28°C for 3 days, with the hyphal growth not yet covering the culture dish, the collected *A. avenae* nematodes were transferred to the culture dish. After another 4– 6 weeks of culture, the nematodes were collected on the lid of the culture dish. They were sterilized with 5% sodium hypochlorite and then centrifuged with 35% sucrose prior to use in the experiment.

### Genome assembly and polishing

We used a variety of software to assemble the genome. First, we used the default parameters of Flye (v2.8.1-b1676) (Kolmogorov et al. 2019) to assemble the ONT data. The genome size was shown to be twice the *k*-mer estimate, but the number of contigs exceeded 600. Then, we used Canu (v2.1) (Koren et al. 2017) to reassemble the genome. To separate homologous chromosomes, we used the parameters corOutCoverage = 200 and “batOptions = -dg 3 -db 3 -dr 1 -ca 500 -cp 50”. After the initial assembly with ONT long reads, we polished the genome using a two-step strategy. First, the draft assembly was polished with gcpp (SMRT Link, PacBio) using PacBio HiFi reads. Second, to further improve base-level accuracy, we applied five iterative rounds of polishing with Illumina paired-end reads to further improve base-level accuracy by using Pilon (Walker et al. 2014). This strategy substantially reduced sequencing errors and ensured a high-quality genome assembly.

### 3D genome construction

To reconstruct the 3D chromosomal maps of each haplotype, we used HiC-Pro (Servant et al. 2015) to generate interaction matrices for each haplotype's nine chromosomes at resolutions of 1 Mb, 500 kb, 100 kb, 40 kb, and 10 kb. Next, we applied miniMDS (Rieber and Mahony 2017) to calculate relative distances within each chromosome at 10-kb resolution and between chromosomes at 100-kb resolution. miniMDS was used to convert the contact frequency matrix into relative positions in a three-dimensional Euclidean coordinate system. The higher the contact frequency, the closer the three-dimensional distance between the fragments. Finally, the inferred 3D coordinate data were imported into Mayavi software (https://github.com/enthought/mayavi) to generate a rotatable and scalable three-dimensional chromosome spatial model.

### Collinearity and genomic variation analysis

The syntenic gene pairs between homologous chromosomes were identified by using JCVI pipeline (Wang et al. 2012). Whole-genome alignment was conducted between the assembled two haploid genome using minimap2 (Li 2018) with the parameters “-ax asm5 ‐‐eqx”. Next, we employed syri (v1.7.0) (Goel et al. 2019) to identify collinear regions, structure variation (inversions, translocations, and duplications), insertion and deletion mutations, and single nucleotide polymorphisms (SNPs). Finally, the plotsr software (Goel and Schneeberger 2022) was employed to visualization.

### Functional annotation and *K*_a_/*K*_s_ calculation

The KEGG and GO functional annotation was performed by using eggNOG-mapper (Huerta-Cepas et al. 2017). The Pfam conserved domain prediction was performed by using pfam_scan.pl (Mistry et al. 2021). The *K*_a_, *K*_s_, and *K*_a_/*K*_s_ values between syntenic gene pairs were calculated by using TBtools-II (Chen et al. 2020). To minimize artifacts due to small denominators, pairs with *K*_s_ < 0.01 were excluded. GO enrichment analysis was performed using the “GO Enrichment” function of TBtools-II.

### Telomeric element identification

We used Tandem Repeats Finder (Benson 1999) to identify tandem repeats on the chromosomes. From the results, we found the classic telomeric repeat sequence “TTAGGC” on the chromosomes. We then used a Python script (available in the Supplemental Code) to count occurrences of the forward tandem repeat “TTAGGCTTAGGC” and the reverse tandem repeat “GCCTAAGCCTAA”. Finally, we visualized the results with RIdeogram (Hao et al. 2020), where each triangle represents two occurrences of the telomeric repeat sequence.

### RNA-seq analysis and expression profile determination

For differentially expressed genes (DEGs) analysis, we used HISAT2 (Kim et al. 2019) to align the reads to each haploid genome, and then SAMtools (Li et al. 2009) was employed to convert the SAM files to BAM files and sort them. HTSeq-count (v0.13.5) (Anders et al. 2015) was then used to calculate the count values of gene expression for each gene. Differential expression analysis was performed using DESeq2 (v1.28.1) (Love et al. 2014). Raw read counts were modeled with a negative binomial distribution, and *P*-values were adjusted for multiple testing using the Benjamini–Hochberg procedure. Genes/alleles with an adjusted *P*-value < 0.05 and an absolute log_2_ fold change >1 were considered differentially expressed. Expression profile analysis was performed using Mfuzz (v2.48.0) (Kumar and Futschik 2007), setting the membership value threshold to greater than 0.4. Gene expression data meeting these criteria were subsequently visualized using pheatmap (v1.0.12). All replicates of transcriptomic data from various developmental stages of *A. avenae*, as well as under desiccation and cold treatments, were used to calculate transcripts per million (TPM) values for each gene's expression at each specific stage by using STAR (Dobin et al. 2013) and RSEM (Li and Dewey 2011).

### *Aap5cs* phylogenetic analysis

The *P5CS* gene sequences of plants and insects were downloaded from NCBI Gene (https://www.ncbi.nlm.nih.gov/gene/). The *P5CS* genes of nematodes were identified by using the *A. avenae Aap5cs* gene as a query in a BLAST search on the WormBase ParaSite web site to find homologous sequences in other nematodes. All genes were then combined and aligned using MUSCLE (Edgar 2004), with nonconserved sequences trimmed using trimAl (Capella-Gutiérrez et al. 2009). A phylogenetic tree was constructed by using FastTree (Price et al. 2010) with parameters “-lg -gamma -slow” and visualized using iTOL. Additionally, the presence of *lea-1*, *tps-1*, and *sod-3* genes in nematodes was determined by using the corresponding genes from *A. avenae* and *C. elegans* as queries in a BLAST search on the WormBase ParaSite web site. Species with homologous genes were selected and marked on the *P5CS* phylogenetic tree.

### *Aap5cs* and *Aatps-1* knockdown

RNAi was performed following our previously established protocol in *D. destructor* (Chen et al. 2025). Briefly, high concentrations of dsRNA were synthesized using a T7 in vitro transcription kit and subsequently diluted to a final concentration of 1 µg/µL, with approximately 5000 mixed stage nematodes included in each reaction system. After RNAi treatment, a subset of nematodes was subjected to desiccation tolerance assays, and the remaining individuals were used for RT-qPCR to assess the knockdown efficiency of the target gene.

### ROS detection

The ROS level in nematodes was detected using the Bio-Tech Reactive Oxygen Species Detection kit S0033S (DCFH-DA method). The DCFH-DA probe was diluted 1.5:1000 in nuclease-free ddH_2_O. Fluorescence images were taken using an Olympus BX63 fully motorized microscope with an excitation wavelength of 502 nm and an emission wavelength of 530 nm. ImageJ was used to calculate the mean fluorescence intensity. The threshold was set to 40 based on the photo with the strongest fluorescence intensity. The fluorescence intensity of the negative control could not be detected under the same setting. To compare the difference, the mean fluorescence intensity of the negative control was artificially defined as 1–3. Each experiment was performed with three independent biological replicates.

More detailed experimental procedures are provided in Supplemental Methods.

## Data access

The PacBio, ONT, Hi-C, and Illumina sequence data generated in this study have been submitted to the NCBI BioProject database (https://www.ncbi.nlm.nih.gov/bioproject/) under accession number PRJNA1191536. The stage-specific RNA-seq data and mixed-stage Iso-Seq data generated in this study have been submitted to the NCBI BioProject database under accession number PRJNA1182914. The genome assembly and annotation files have been submitted to Genome Warehouse of the China National Center for Bioinformation (https://ngdc.cncb.ac.cn/gwh/) under accession number GWHHAIJ00000000.1, and are also available on our laboratory website (https://bmb.hzau.edu.cn/info/1031/1543.htm).

## Supplemental Material

Supplement 1

Supplement 2

Supplement 3

Supplement 4
