# Supplementary material for: Haplotype-resolved telomere-to-telomere genome of Aphelenchus avenae implicates P5CS in nematode desiccation stress response
Source: Genome Res. 2026 Apr;36(4):726–39. doi: 10.1101/gr.281016.125 (PMC13138009; doi:10.1101/gr.281016.125)
Supplement: Supplement 1 [file Supplemental_material.pdf]

Supporting Information for

Haplotype-resolved telomere-to-telomere genome of *Aphelenchus avenae* implicates *P5CS* in nematode desiccation stress response

Yali Zhang†, Yayi Zhou†, Yangyang Chen, Xueyu Wang, Boyan Hu, Shahid Siddique, Romnick A. Latina, Veronica Casey, Dexin Bo, Yucheng Liao, Min Zhang, Ming Sun, Fengjuan Zhang*, and Dadong Dai*

Corresponding author: *Dadong Dai and Fengjuan Zhang
Email: dddai@ucdavis.edu; zhangfengjuan159@163.com

†These authors contributed equally to this work.

This PDF file includes:

Supplemental Methods pages 2-10
Supplemental Figures pages 11-34

Supplemental Methods

DNA and RNA extraction and library construction

For PacBio and ONT sequencing, genomic DNA was extracted from approximately 500,000 mixed-stage nematodes using our previously modified CTAB-based nematode DNA extraction method (Dai et al. 2023), and at least 8 μg of high-quality DNA was used for library construction and sequencing. PacBio used the sequel I platform for sequencing, and ONT used PromethION flow cells. The pore version used was R9.4.1, and the PromethION release version was 19.05.1. For Illumina short-reads sequencing, 100ng DNA was used to construct the library and the samples were sequenced on Illumina NovaSeq platform with a 300–500 bp insert size. RNA extraction was performed using the TransZol Up Plus RNA Kit (TransGen Biotech, Beijinng, China). The RNA-seq data generated in this study include cold-treated mixed-stage samples (three biological replicates) using a conventional transcriptome sequencing approach, and stage-specific samples (J2, J3/J4, male, and female, each with 2–4 biological replicates, under normal growth conditions) using single-nematode transcriptome sequencing (3-5 nematodes per replicate). In addition, previously published datasets (RH0, RH40, RH85, RH97, RH100), each with three replicates of mixed-stage nematodes, were downloaded from SRA database (PRJNA236622) and incorporated into our analysis. The RNA-seq library was constructed by using VAHTS Universal V10 RNA-seq Library Prep Kit for Illumina (NR606-01). The single-nematode RNA-seq library construction was performed as we described before (Wang et al. 2024), following the protocol of sensitive highly multiplexed single-cell RNA-seq (Smart-seq2).

Hi-C library construction and sequencing

The Hi-C library construction was performed following our previous method. Briefly, after the mixed stage *A. avenae* nematodes were collected, they were disinfected with 5% sodium hypochlorite (sigma) for 5 minutes, and then impurities were removed by centrifugation with 35% sucrose. Then, 500ul of PBS was used to suspend the nematodes, and formaldehyde was added to a final concentration of 1.5%. DNA was cross-linked at room temperature for 30 minutes. After which, glycine was added to a final concentration of 0.75M to terminate the reaction. The nematode samples were flash-frozen with liquid nitrogen and thoroughly ground After grinding the nematode sample, MboI was used for digestion for 7 hours, followed by biotin addition, connection, uncross-linking, DNA interruption, and T4 DNA polymerase to remove the biotin that was not correctly connected to the end. After pulling down the biotin-labeled DNA with streptavidin affinity magnetic beads, the library was constructed and sequenced on an Illumina NovaSeq platform with 150 bp paired-end reads.

Genome survey

We used Illumina short-read data to evaluate the genome size, ploidy, and heterozygosity of the *A. avenae* genome through *k*-mer analysis. First, we performed *k*-mer counting using kmc (Kokot et al. 2017) with the parameters "-k21 -t16 -m64 -ci1 -cs10000 -fm". Next, we used kmc_tools to further process the *k*-mer counting results to generate a *k*-mer frequency histogram. We then employed the cutoff function in smudgeplot.py to calculate the lower and upper *k*-mer frequency cutoffs, which were used to define the range of *k*-mer frequencies of interest for subsequent analysis to help identify ploidy and other genomic characteristics. Based on these cutoff values, we used kmc_tools transform to export *k*-mers that met the defined frequency range (as specified by L and U) to a dump file in text format. Using the resulting "dump" file, we applied the hetkmers function in smudgeplot.py (Ranallo-Benavidez et al. 2020) to estimate the

genome's ploidy. Finally, with the identified ploidy and *k*-mer frequency data, we used GenomeScope2 (Ranallo-Benavidez et al. 2020) to assess genome size and heterozygosity.

Chromosome mounting and genome collapse detection

The Hi-C data were used to mount the contigs to the chromosome. Firstly, the Hi-C reads were mapped to the assembled contigs using Juicer (v1.5.7) pipeline (Durand et al. 2016b). The “merged_nodups.txt” file were used as an input file for 3d-dna (v180419) pipeline (Dudchenko et al. 2017) with parameters “-r 0” to construct the chromosome according to Hi-C signal. The output results were then imported into JuiceBox (v1.13.01) (Durand et al. 2016a) for visualization, we manually corrected contig positions according to Hi-C contact maps. Specifically, contigs showing strong and unambiguous interaction signals with distant chromosomal regions were reassigned, and those with inverted signal patterns were reoriented accordingly. Two chromosomes with homologous chromosome signals were manually aligned together and sorted by size. After genome assembly, we use BUSCO (v5.7.1) genome/protein module to evaluate the completeness of genome/protein by using nematoda_odb10 as a database. For clarity, hap1 and hap2 were distinguished only by chromosome size during assembly partitioning. Thus, the observed BUSCO completeness differences between hap1 and hap2 are not interpreted as true haplotype-specific variation, but rather as a consequence of this separation approach. To detect the chromosome collapse, the Illumina short-reads were aligned to the genome by using BWA (v0.7.17-r1188) (Li and Durbin 2009). Subsequently, BEDTools (v2.29.2) (Quinlan and Hall 2010) coverage was used to count the average read depth within a 100 kb window on the genome. We divided the genome into two haploid genomes (hap1 and hap2) based on Hi-C signals.

Protein coding gene annotation

To annotate the genome of *A. avenae*, we generated Iso-Seq data of mix stage and RNA-seq data of second-stage juveniles (J2), third/fourth-stage juveniles (J3/J4), female, and male. Additionally, we obtained transcriptome data under dehydration and 4°C stress for 2 hours in the mix stage. First, we soft-masked the repetitive sequences in the genome using RepeatMasker (Tarailo-Graovac and Chen 2009). Then, we aligned the RNA-seq data to the masked genome with HISAT2 (Kim et al. 2019), and the resulting BAM files were used as input for BRAKER3 (Gabriel et al. 2024) to generate the annotation file braker.gff3. Simultaneously, the aligned BAM files were used with TransDecoder (https://github.com/TransDecoder/) and StringTie (v2.1.1) (Pertea et al. 2015) for annotation, producing stringtie.gff3. Additionally, we performed a *de novo* assembly of the transcriptome data using Trinity (v2.15.2) (Grabherr et al. 2011), resulting in Trinity.fasta. Full-length isoforms were obtained through IsoSeq3 software (https://github.com/PacificBiosciences/IsoSeq), and redundant sequences were removed using CD-HIT-EST (v4.8.1) (Fu et al. 2012). The Iso.fasta was combined with Trinity.fasta and then used as input for PASA (2.5.3) (Haas et al. 2008) to predict coding genes, generating the file pasa_assemblies.gff3. Finally, we integrated the three GFF3 files using EVidenceModeler (Haas et al. 2008) to produce a single GFF3 file, representing the final annotation.

TE, HGT, and secreted protein prediction

Transposable elements (TE) were detected using the EDTA (v2.2.0) (Ou et al. 2019) pipeline with the parameters "--overwrite 1 --force 1 --sensitive 1 --anno 1 --evaluate 1". Candidate HGT genes were predicted by using AvP pipeline (Koutsovoulos et al. 2022) with uniref90 database, and the candidate gene with HGT or HGT-NT label was considered as HGT gene. When we use AvP, Ingroup selects "33208: Metazoa" and

EGP selects "33283: Tylenchomorpha". We used SignalP5.0 (Almagro Armenteros et al. 2019) to predict signal peptides for all proteins and then used tmhmm-2.0c (Krogh et al. 2001) with model2.0 to predict transmembrane domains for all genes predicted to have signal peptides, and those proteins with secretory signal peptides but no transmembrane domains were considered as putative secreted proteins.

Allele imbalance expression gene definition

For comparisons of gene expression across different developmental stages, the mean expression of all biological replicates was taken as the gene's expression value at that stage. Allele imbalance expression (AIE) was defined by assessing the significance of expression differences between two alleles across all biological replicates at the same stage, with a p-value threshold of 0.05 to determine AIE. For significance testing of expression differences within a group of alleles at the same stage, a t-test was applied. For assessing differences between J2 to female stages and under different desiccation conditions, a two-way ANOVA was used.

Classification of homologous genes

We classified high-confidence homologous genes based on similarity as follows: 1) those with completely identical amino acid sequences and coverage; 2) those with identical coverage but differing amino acid sequences; 3) genes where hap1 has 100% coverage but hap2 has less than 100%; 4) genes where hap2 has 100% coverage but hap1 has less than 100%; 5) homologous genes with 90%-100% coverage; 6) homologous genes with 80%-90%, and 7) and genes with less than 80% coverage. According to the classification criteria above, the corresponding numbers of genes are as follows:

gene number	identity	coverage of gene from hap1	coverage of gene from hap2
1027	100%	100%	100%
7392	< 100%	100%	100%
2686	> 75%	100%	< 100%
1979	> 75%	< 100%	100%
2059	> 75%	90% ≤ coverage < 100%	90% ≤ coverage < 100%
227	> 75%	80% ≤ coverage < 90%	80% ≤ coverage < 90%
481	> 75%	70% ≤ coverage < 80%	70% ≤ coverage < 80%

In vitro RNAi protocol

Primers with T7 promoter sequences were designed for the two selected target genes, respectively, as follows:

ds*Aap5cs*-F: TAATACGACTCACTATAGGGCTGAAAATCAGTCGTACCGCT

ds*Aap5cs*-R: TAATACGACTCACTATAGGGCTTGATCGACTCTGGACAGA

ds*Aatps-1*-F: TAATACGACTCACTATAGGGACTCCCTCAACGACTCT

ds*Aatps-1*-R: TAATACGACTCACTATAGGGTTGGAGACGTTGATCACG

dsGFP-R (control): TAATACGACTCACTATAGGGGGTCTGCTAGTTGAACGCTTCC

dsGFP-F (control): TAATACGACTCACTATAGGGGAGTGCCATGCCCGAAGGTTA

RNA Interference Protocol for Mixed-Stage *A. avenae* Nematodes:

Total RNA was extracted from mixed-stage *A. avenae* nematodes and reverse-transcribed into cDNA. The cDNA was used as a template for amplifying the target gene using the above primers. The PCR product was purified using the OMEGA Gel Extraction kit (eluted in RNase-free ddH$_2$O), and the DNA concentration was adjusted to 100 ng/μL. Double-stranded RNA (dsRNA) was then synthesized *in vitro* using the Vazyme T7 High Yield RNA Transcription kit (Cat: TR101-01).

Interference system setup:

1) Add dsRNA targeting the gene of interest to a final concentration of 1 μg/μL.

2) Include approximately 5000 mixed-stage nematodes in the system.

3) For each 100 μL reaction system, add the following:

5 μL of 1M octopamine

0.3 μL of 1M spermidine

2 μL of 1% Triton X-100

4) Incubate at room temperature with gentle rotation at 15 rpm/min for 56 hours.

Desiccation Time Testing: Following RNA interference, ds*Aap5cs-1* showed cuticle shrinkage at 11 minutes and 30 seconds, while ds*Aatps-1* exhibited cuticle shrinkage at 12 minutes. Based on these results, desiccation treatments were set at 6 and 12 minutes to examine post-desiccation phenotypes in nematodes. The recovery of nematodes was standardized by quickly rehydrating them, followed by observation after 3 minutes.

ROS detection

Preliminary Experiment: A total of 1000 mixed-stage *A. avenae* nematodes were placed on a 3 μm microporous membrane, and water was removed using vacuum filtration. The nematodes were then subjected to 40% relative humidity at 25°C in an incubator. Survival time was monitored, with nematode cuticles showing shrinkage after approximately 24 minutes. Rapid rehydration was performed, and recovery was assessed at 3 minutes, during which ~15% of the nematodes showed signs of activity. After 90 minutes, ~65% of the nematodes recovered (further extension of recovery time did not increase the percentage of recovered nematodes).

Experimental Design:

Treatment Group: Following RNA interference, the mix stage nematodes were desiccated for a specified duration (6, 12, and 24 minutes, the same method with

preliminary experiment). A subset was rehydrated for 3 minutes, and the number of recovered nematodes was counted. Another subset was quickly ground in liquid nitrogen, incubated with 100 μL of diluted DCFH-DA probe at 37°C for 20 minutes, and then imaged using an Olympus BX63 fluorescence microscope. Nematodes that were not subjected to desiccation after RNAi treatment were processed and imaged using the above protocol to rule out the possibility of ROS accumulation caused by the RNAi process.

H_2O Control Group: Nematodes not treated with RNAi were desiccated for specified durations (6, 12, and 24 minutes). A subset was rehydrated in water for 3 minutes, and the number of recovered nematodes was counted. Another subset was quickly ground in liquid nitrogen, incubated with 100 μL of diluted DCFH-DA probe at 37°C for 20 minutes, and then imaged.

Positive Control Group: Nematodes were pre-treated with the positive control reagent Rosup at 37°C for 20 minutes. Following liquid nitrogen grinding, they were incubated with 100 μL of diluted DCFH-DA probe at 37°C for 20 minutes and imaged.

Negative Control Group: No treatments were applied. Nematodes were quickly ground in liquid nitrogen without adding any probe to exclude autofluorescence signals.

Reference:

Almagro Armenteros JJ, Tsirigos KD, Sonderby CK, Petersen TN, Winther O, Brunak S, von Heijne G, Nielsen H. 2019. SignalP 5.0 improves signal peptide predictions using deep neural networks. *Nat Biotechnol* **37**: 420-423.

Dai D, Xie C, Zhou Y, Bo D, Zhang S, Mao S, Liao Y, Cui S, Zhu Z, Wang X. 2023. Unzipped chromosome-level genomes reveal allopolyploid nematode origin pattern as unreduced gamete hybridization. *Nat Commun* **14**: 7156.

Dudchenko O, Batra SS, Omer AD, Nyquist SK, Hoeger M, Durand NC, Shamim MS, Machol I, Lander ES, Aiden AP et al. 2017. De novo assembly of the *Aedes aegypti* genome using Hi-C yields chromosome-length scaffolds. *Science* **356**: 92-95.

Durand NC, Robinson JT, Shamim MS, Machol I, Mesirov JP, Lander ES, Aiden EL. 2016a. Juicebox Provides a Visualization System for Hi-C Contact Maps with Unlimited Zoom. *Cell Syst* **3**: 99-101.

Durand NC, Shamim MS, Machol I, Rao SS, Huntley MH, Lander ES, Aiden EL. 2016b. Juicer Provides a One-Click System for Analyzing Loop-Resolution Hi-C Experiments. *Cell Syst* **3**: 95-98.
Fu L, Niu B, Zhu Z, Wu S, Li W. 2012. CD-HIT: accelerated for clustering the next-generation sequencing data. *Bioinformatics* **28**: 3150-3152.
Gabriel L, Bruna T, Hoff KJ, Ebel M, Lomsadze A, Borodovsky M, Stanke M. 2024. BRAKER3: Fully automated genome annotation using RNA-seq and protein evidence with GeneMark-ETP, AUGUSTUS, and TSEBRA. *Genome Res* **34**: 769-777.
Grabherr MG, Haas BJ, Yassour M, Levin JZ, Thompson DA, Amit I, Adiconis X, Fan L, Raychowdhury R, Zeng Q. 2011. Trinity: reconstructing a full-length transcriptome without a genome from RNA-Seq data. *Nat Biotechnol* **29**: 644.
Haas BJ, Salzberg SL, Zhu W, Pertea M, Allen JE, Orvis J, White O, Buell CR, Wortman JR. 2008. Automated eukaryotic gene structure annotation using EVidenceModeler and the Program to Assemble Spliced Alignments. *Genome Biol* **9**: R7.
Kim D, Paggi JM, Park C, Bennett C, Salzberg SL. 2019. Graph-based genome alignment and genotyping with HISAT2 and HISAT-genotype. *Nat Biotechnol* **37**: 907-915.
Kokot M, Dlugosz M, Deorowicz S. 2017. KMC 3: counting and manipulating *k*-mer statistics. *Bioinformatics* **33**: 2759-2761.
Koutsovoulos GD, Granjeon Noriot S, Bailly-Bechet M, Danchin EGJ, Rancurel C. 2022. AvP: A software package for automatic phylogenetic detection of candidate horizontal gene transfers. *PLoS Comput Biol* **18**: e1010686.
Krogh A, Larsson B, von Heijne G, Sonnhammer EL. 2001. Predicting transmembrane protein topology with a hidden Markov model: application to complete genomes. *J Mol Biol* **305**: 567-580.
Li H, Durbin R. 2009. Fast and accurate short read alignment with Burrows-Wheeler transform. *Bioinformatics* **25**: 1754-1760.
Ou S, Su W, Liao Y, Chougule K, Agda JRA, Hellinga AJ, Lugo CSB, Elliott TA, Ware D, Peterson T et al. 2019. Benchmarking transposable element annotation methods for creation of a streamlined, comprehensive pipeline. *Genome Biol* **20**: 275.
Pertea M, Pertea GM, Antonescu CM, Chang TC, Mendell JT, Salzberg SL. 2015. StringTie enables improved reconstruction of a transcriptome from RNA-seq reads. *Nat Biotechnol* **33**: 290-295.
Quinlan AR, Hall IM. 2010. BEDTools: a flexible suite of utilities for comparing genomic features. *Bioinformatics* **26**: 841-842.
Ranallo-Benavidez TR, Jaron KS, Schatz MC. 2020. GenomeScope 2.0 and Smudgeplot for reference-free profiling of polyploid genomes. *Nat Commun* **11**: 1432.
Tarailo-Graovac M, Chen N. 2009. Using RepeatMasker to identify repetitive elements in genomic sequences. *Curr Protoc Bioinformatics* **Chapter 4**: 4 10 11-14 10 14.
Wang X, Guo Z, Dai D, Xie C, Zhao Z, Zheng J, Sun M, Peng D. 2024. High-resolution transcriptome datasets during embryogenesis of plant-parasitic nematodes. *Sci Data* **11**: 690.

Supplemental Figures

Supplemental_Fig_S1

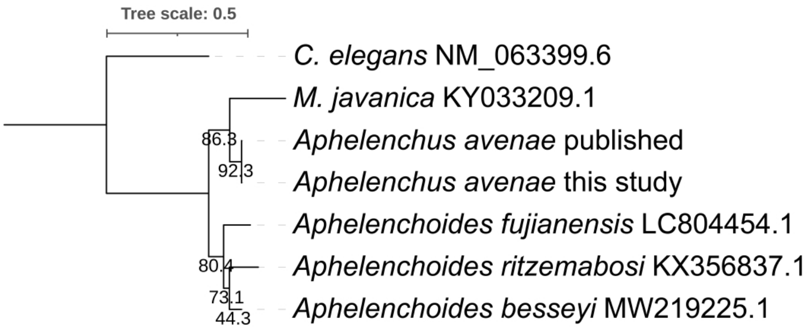

Fig. S1 Phylogenetic tree based on 28S rRNA sequences showing that the nematode used in this study clusters with published *A. avenae* and clearly separates from *Aphelenchoides* species. Accession numbers are shown after species names. The 28S rRNA sequence of the nematode used in this study is located at Chr_5_01: 13615646–13615982, while the published *A. avenae* (GCA_020875895.1) 28S rRNA sequence is located at JNEY01007307.1: 1–334.

Supplemental_Fig_S2

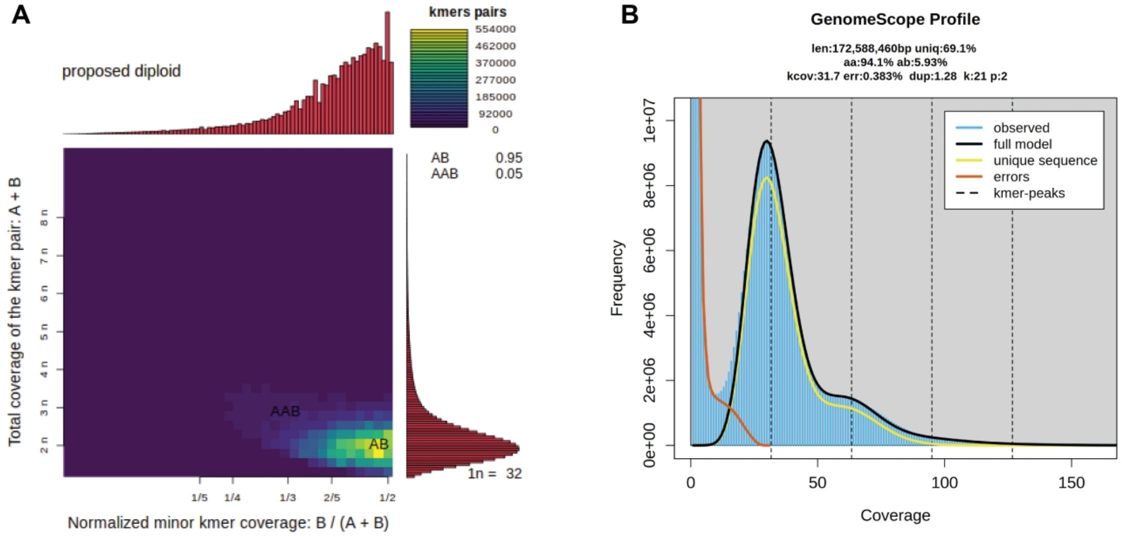

Fig. S2 Evaluation of ploidy, heterozygosity and genome size of the *A. avenae* genome using *k*-mer method. (A) *k*-mer-based ploidy assessment of the *A. avenae* genome shows it to be diploid. **(B)** The estimated haploid genome size of *A. avenae* is 172 Mb, with a heterozygosity of 6%.

Supplemental_Fig_S3

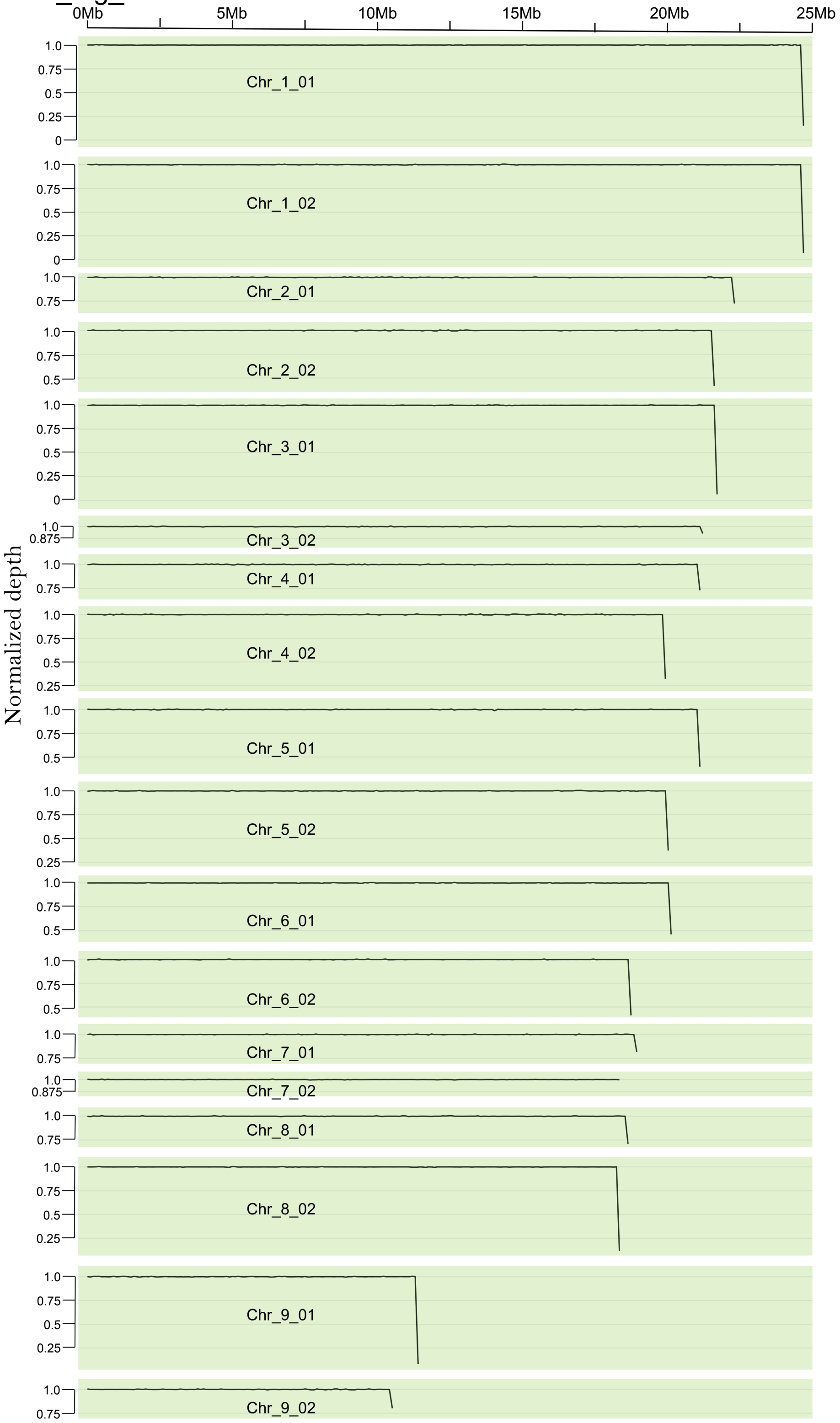

Fig. S3 Average coverage of the *A. avenae* genome evaluated using Illumina data.

Supplemental_Fig_S4

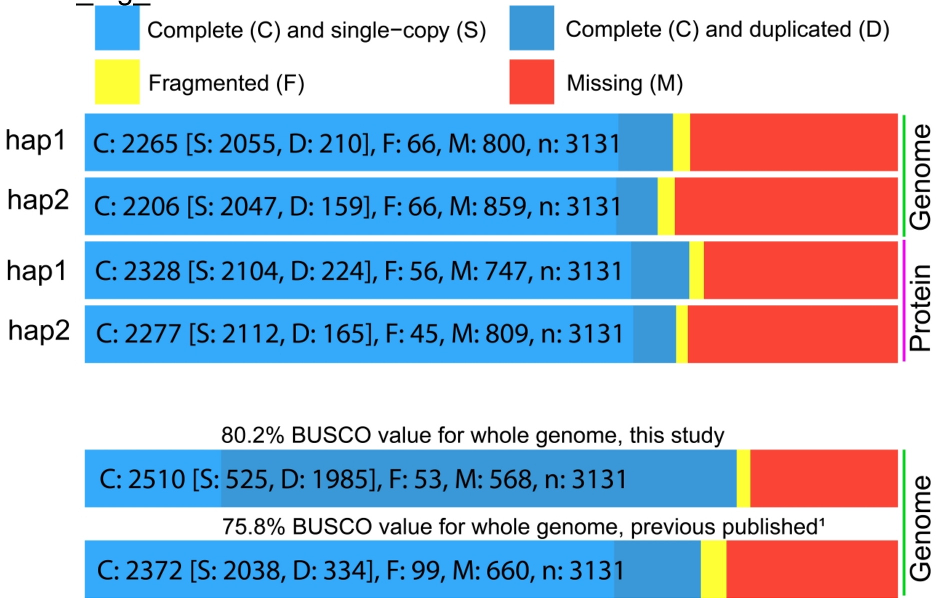

Fig. S4 BUSCO assessment of genomes and protein of *A. avenae.*

[1]Wan X, Saito JA, Hou S, Geib SM, Yuryev A, Higa LM, Womersley CZ, Alam M. 2021. The *Aphelenchus avenae* genome highlights evolutionary adaptation to desiccation. *Commun Biol* 4: 1232.

Supplemental_Fig_S5

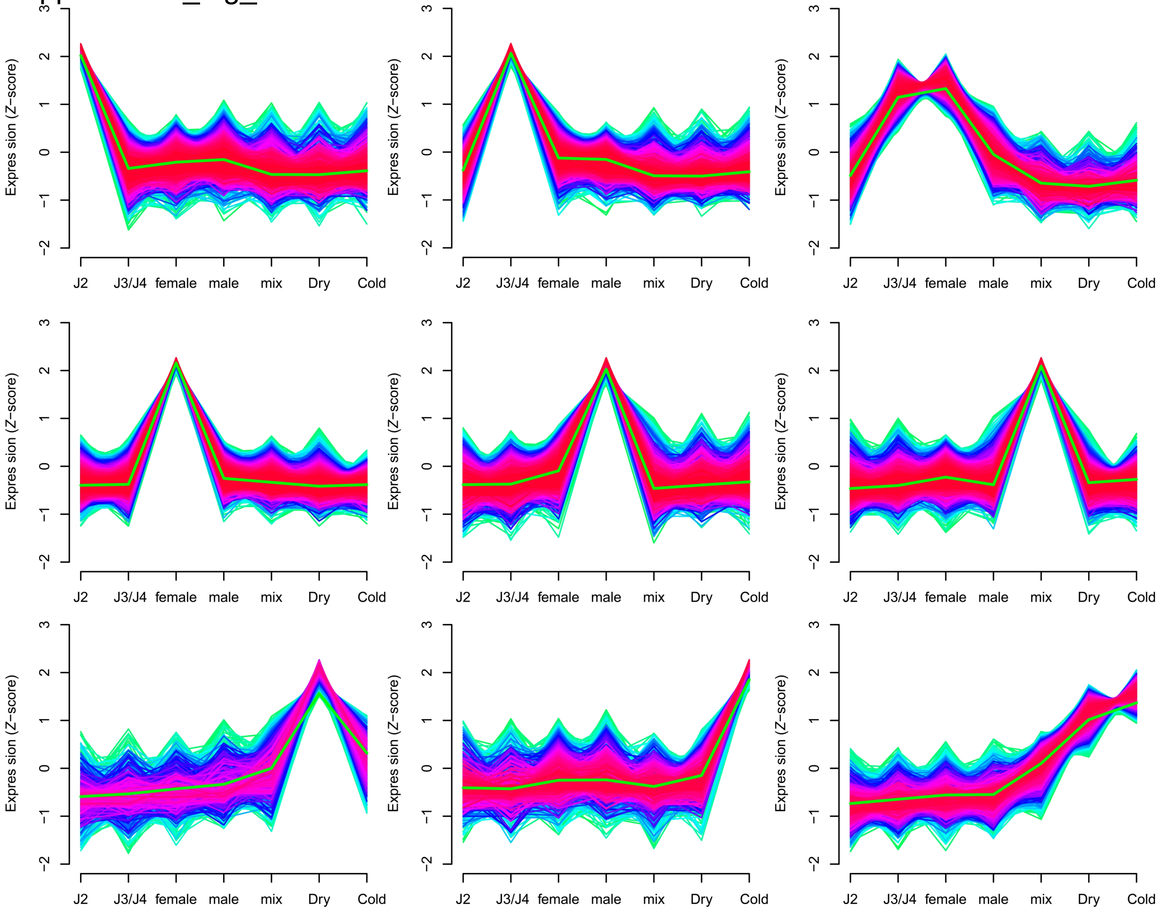

Fig. S5 Gene expression pattern landscape of *A. avenae* during five developmental stages and under drought and cold stress. The results shows that each stage and stress treatment have a set of genes that are specifically highly expressed.

Supplemental_Fig_S6

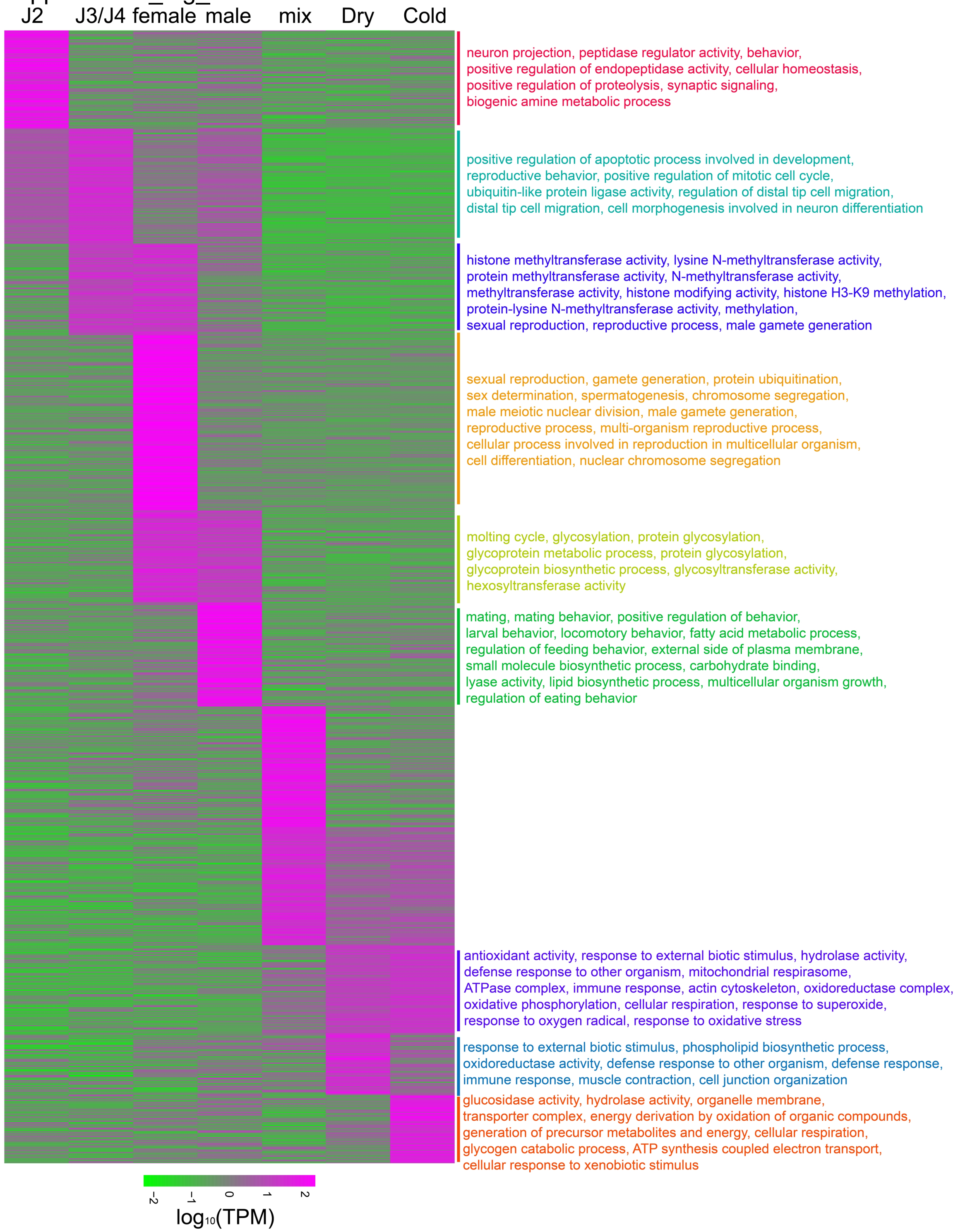

Fig. S6 Transcriptional profiles of *A. avenae* at different stages and environmental stress and corresponding GO functional annotations. Results show that the functional enrichment of genes with high expression are stage- and environmental stress-specific and are often associated with stage-specific biological activity.

Supplemental_Fig_S7

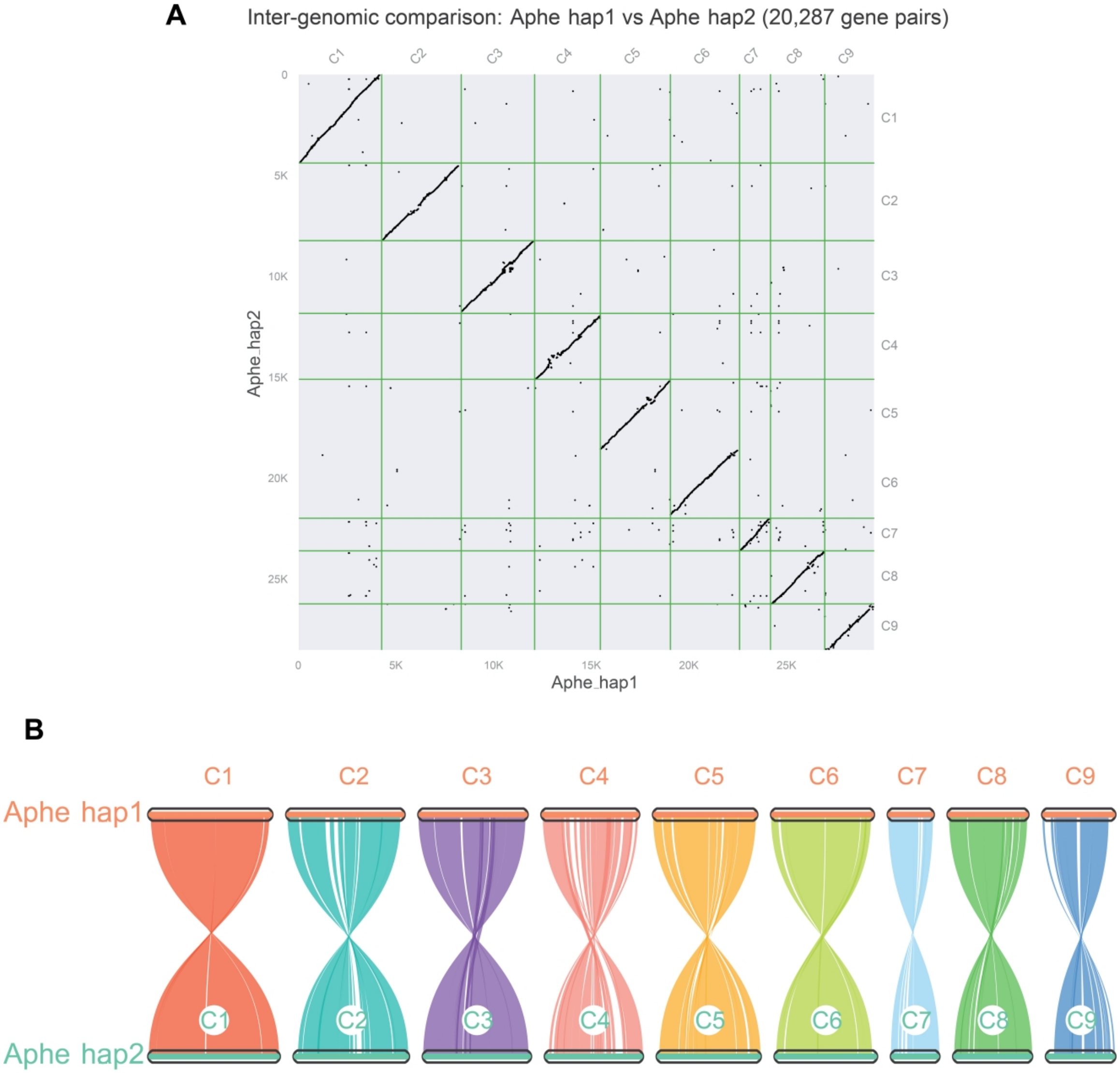

Fig. S7 Analysis of collinearity between two sets of homologous chromosomes of *A. avenae*. (A) The dot plot of collinear genes between hap1 and hap2 of *A. avenae* shows that Chromosome 7 had a small number of collinear genes. (B) The collinear block shows shared collinearity between hap1 and hap2.

Supplemental_Fig_S8

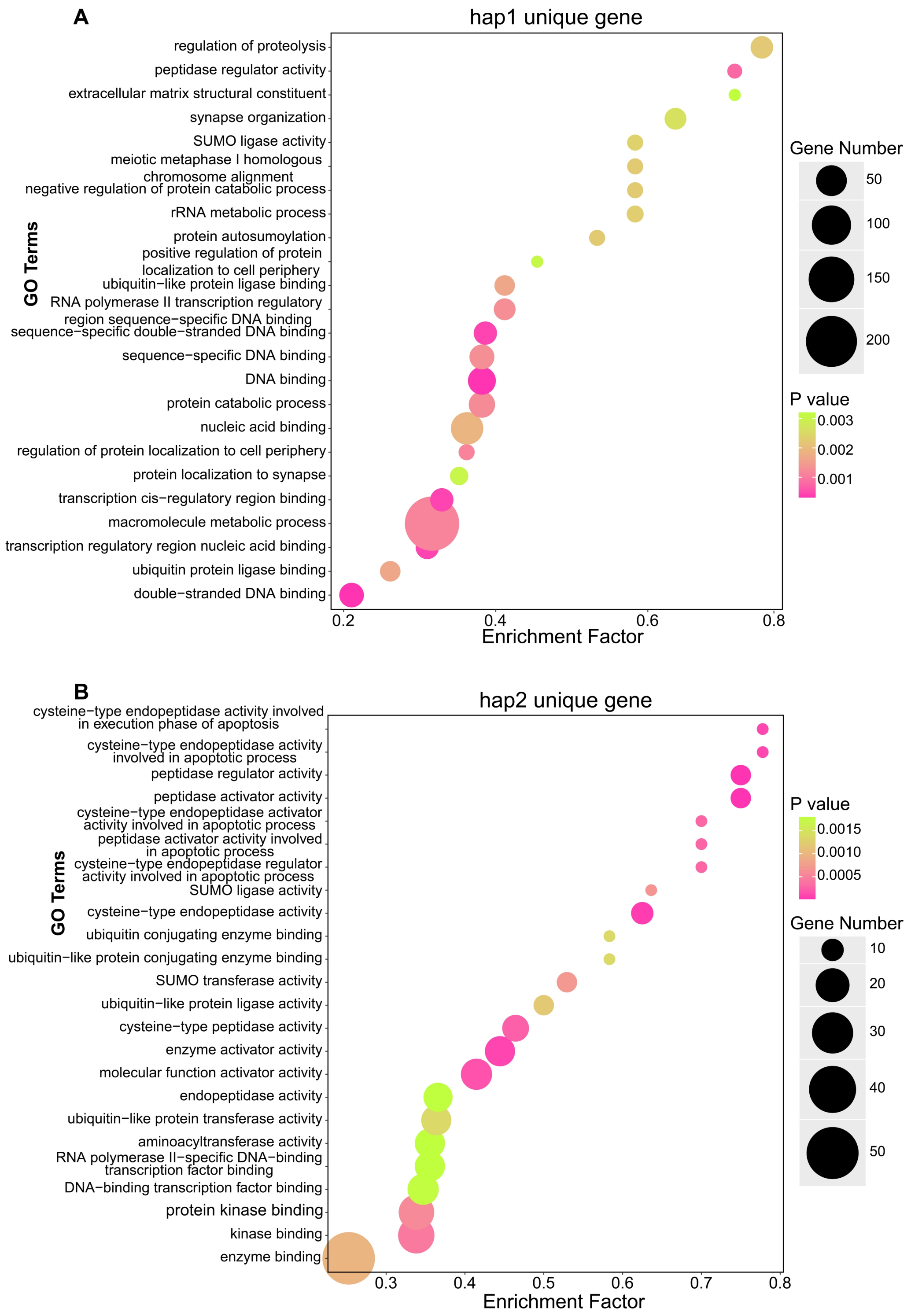

Fig. S8 GO functional enrichment analysis of genes unique to the haplotype genome. (A) GO functional enrichment of hap1-specific genes. **(B)** GO functional enrichment of hap2-specific genes.

Supplemental_Fig_S9

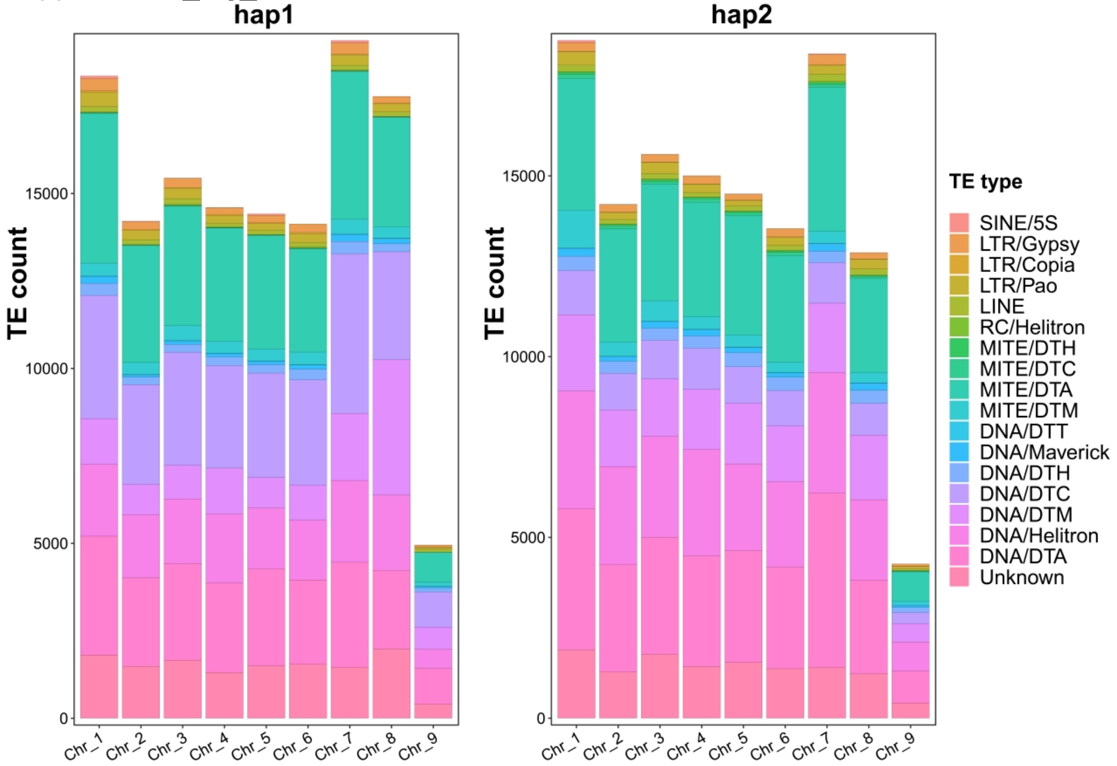

Fig. S9 The number of different TE types on each chromosome of the two-haplotype genome.

Supplemental_Fig_S10

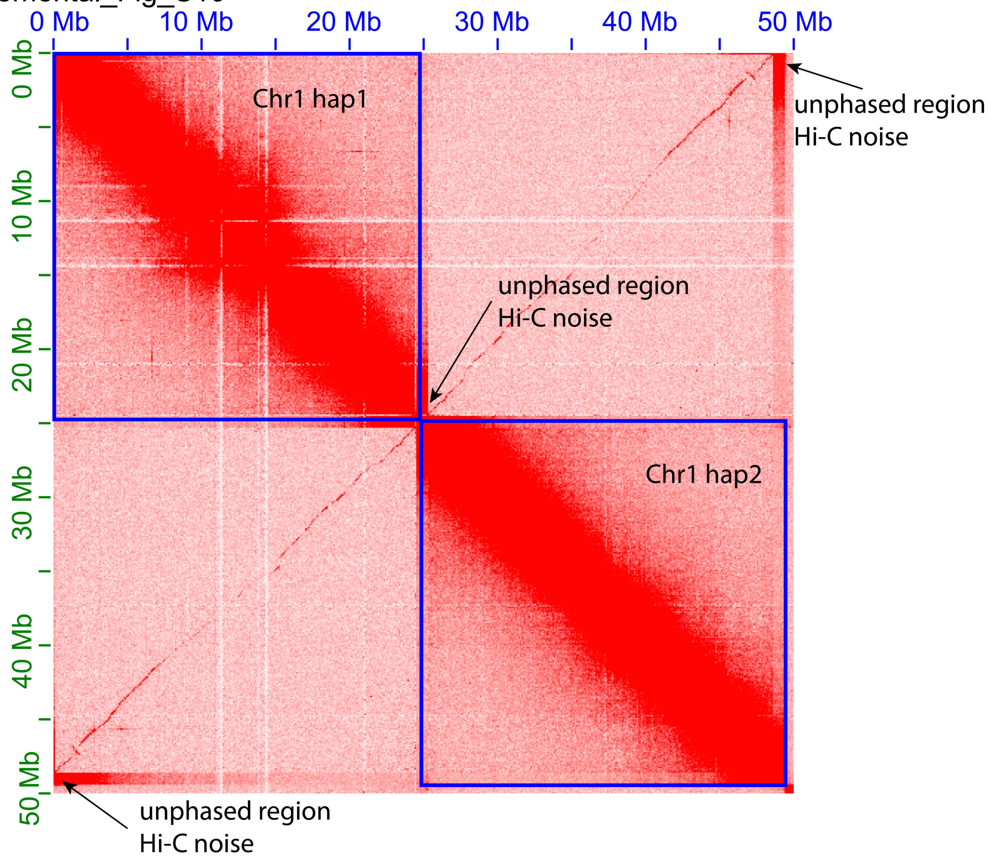

Fig. S10 The assembled genome has collapsed telomeric regions.

Supplemental_Fig_S11

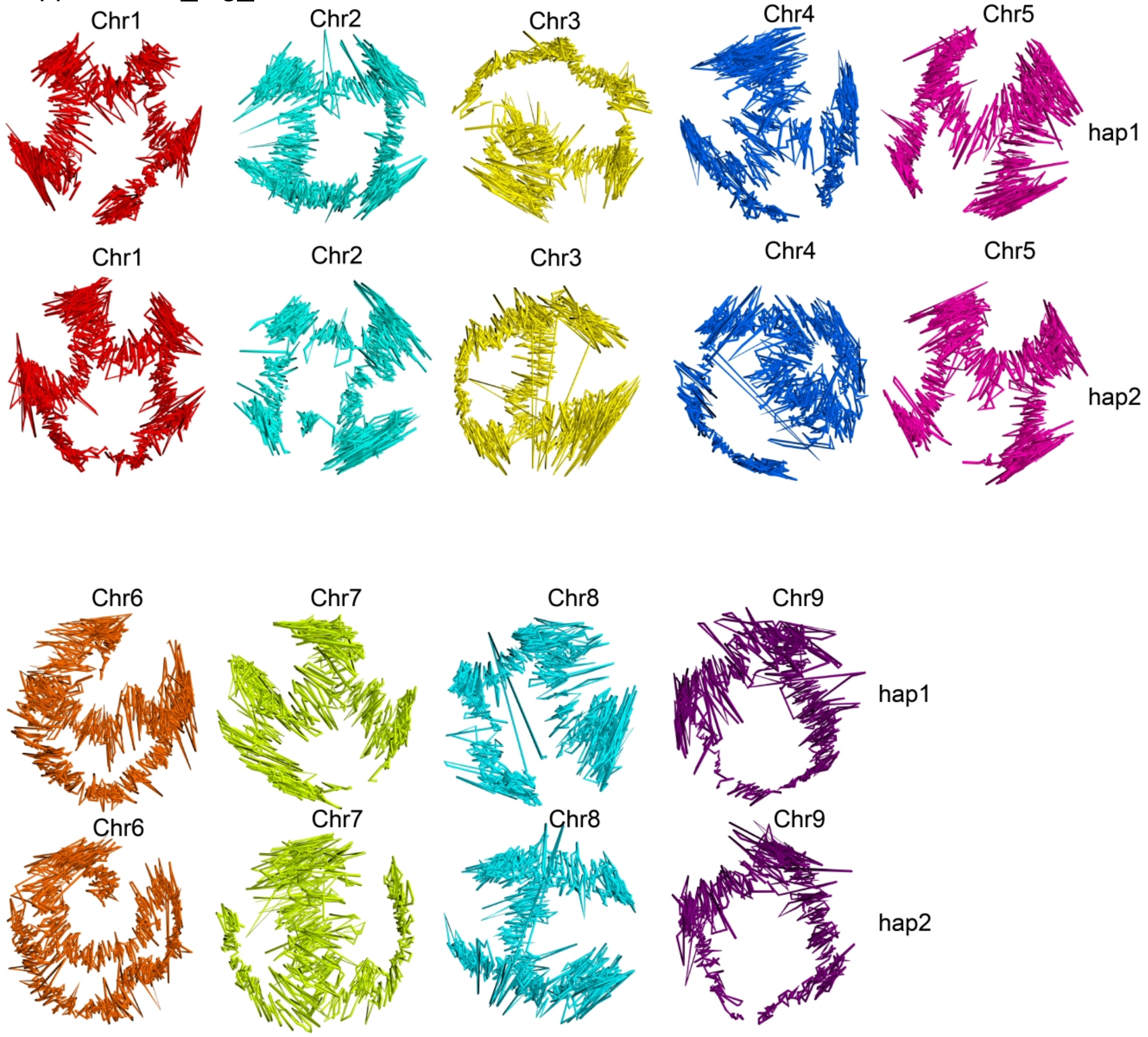

Fig. S11 Hard-masking of non-syntenic regions reshapes chromosomal 3D structures and enhances structural similarity between homologous chromosomes in *A. avenae*.

Supplemental_Fig_S12

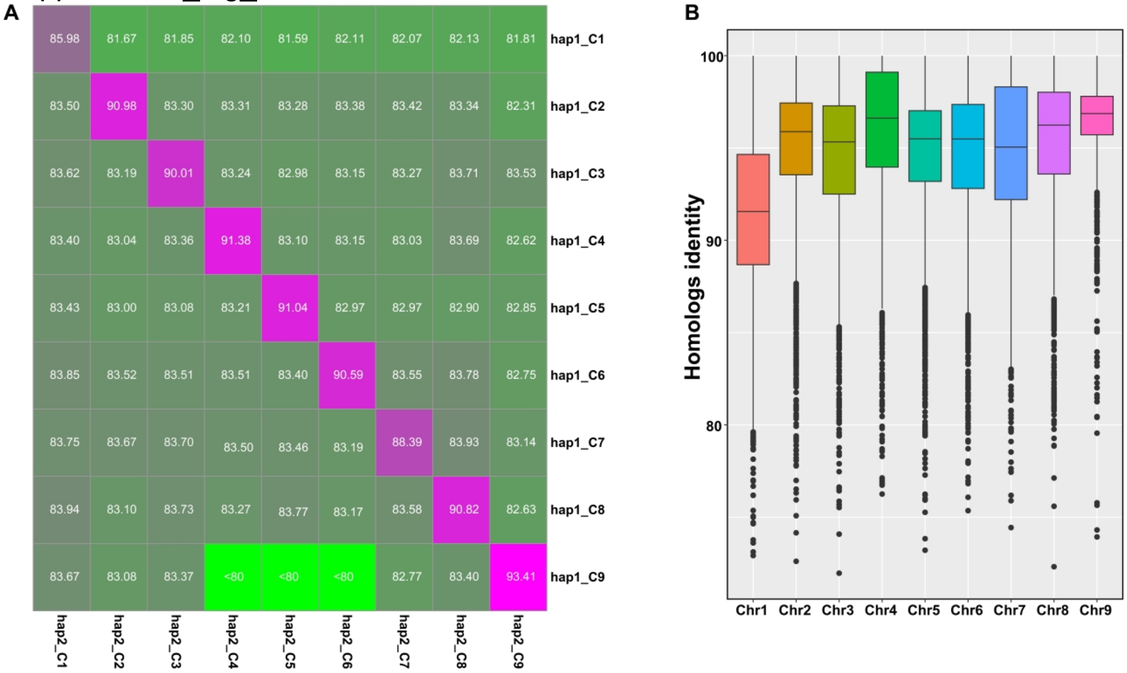

C

Chromosome	Chr1	Chr2	Chr3	Chr4	Chr5	Chr6	Chr7	Chr8	Chr9
Average identity	91.7	95.0	94.4	95.8	94.7	94.6	94.5	95.3	96.4
Variation	4.89	4.06	4.25	4.18	4.01	4.08	4.66	4.11	2.73

Fig. S12 The average and variation in sequence identity between homologous chromosomes and between homologous gene pairs. (A) The average nucleotide identity between *A. avenae* whole genome sequences. **(B)** The nucleotide identity between homologous gene pairs on each pair of homologous chromosomes of *A. avenae*. **(C)** The average nucleotide identity and variation between homolog gene pairs on each pair of chromosomes of *A. avenae*.

Supplemental_Fig_S13

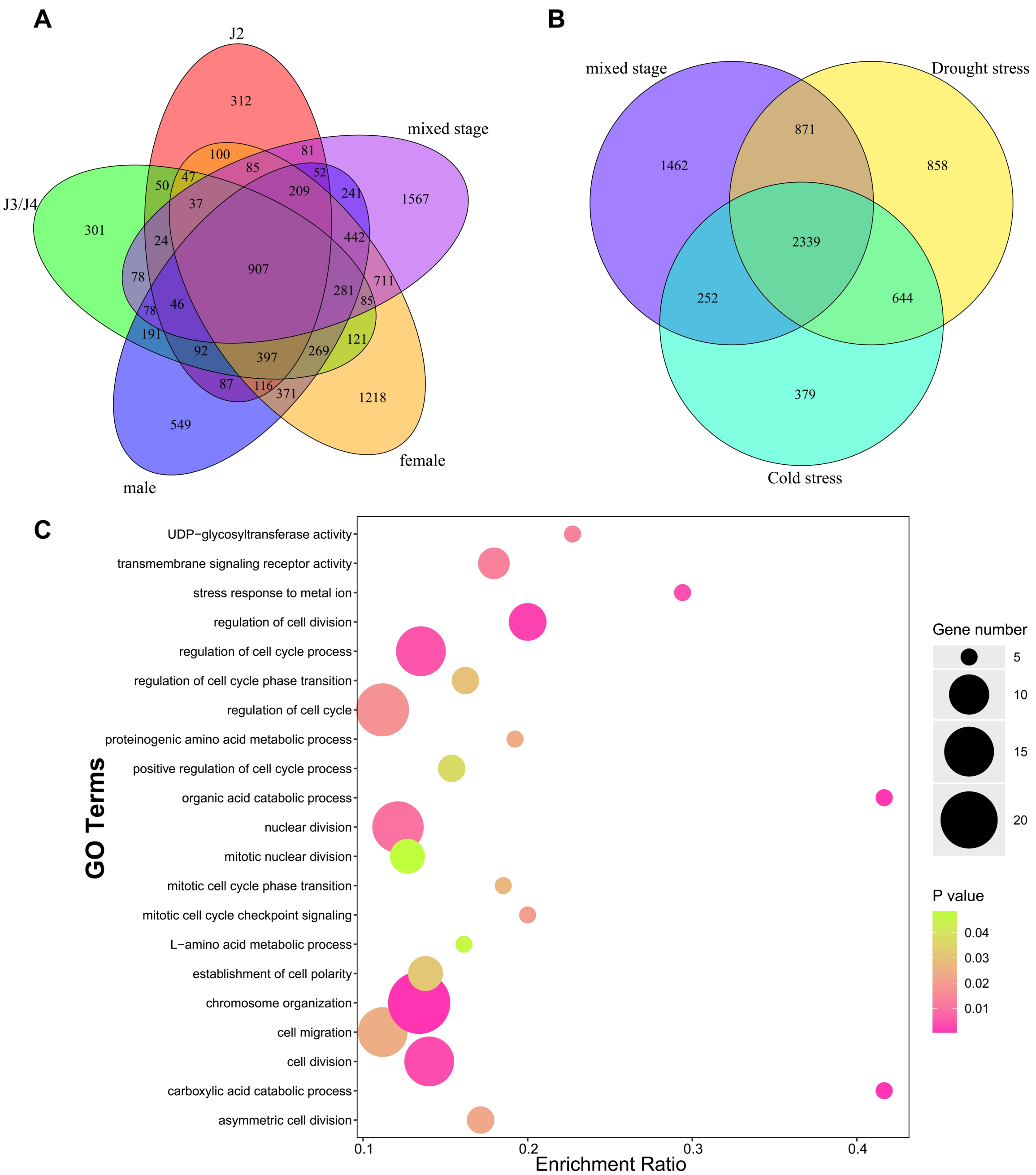

Fig. S13 Differentially expressed alleles (DEAs) at each stage and their overlapping DEAs' GO functional enrichment. (A) Venn diagram of differentially expressed alleles (DEAs) at different developmental stages. Shared genes indicate that the genes are differentially expressed at different developmental stages. **(B)** Venn diagram of DEAs of *A. avenae* mixed stages under desiccation stress, cold stress and normal condition. **(C)** GO functional enrichment of DEAs under desiccation stress.

Supplemental_Fig_S14

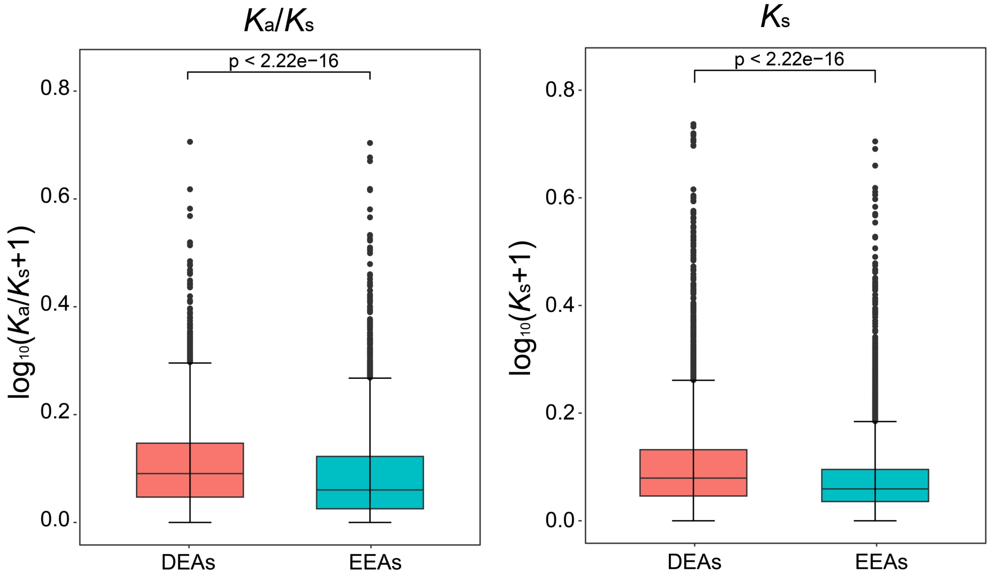

Fig. S14 Comparison of K_a/K_s and K_s values between differentially expressed alleles (DEAs) and equivalently expressed alleles (EEAs). In the box plot, the central line of shows the median, the box represents the 25% and 75% percentiles, and the whiskers are the 1.5x interquartile range beyond the box.

Supplemental_Fig_S15

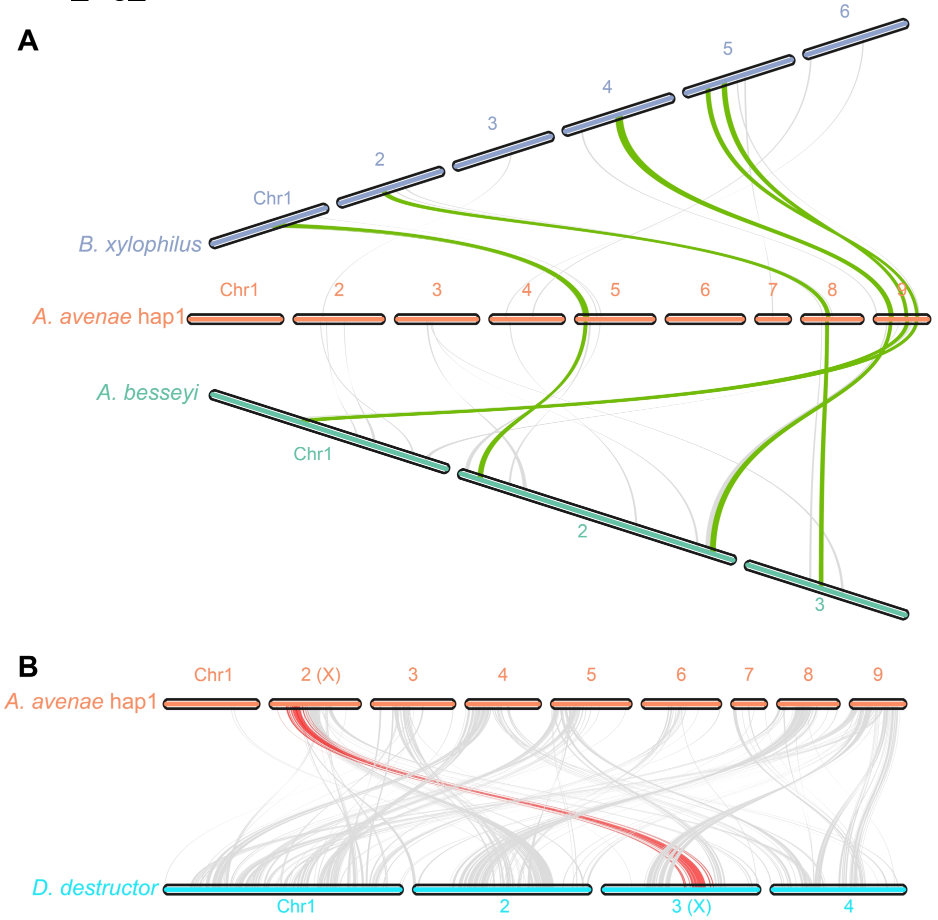

Fig. S15 Comparative chromosomal collinearity between *A. avenae* and several other migratory plant-parasitic nematodes. (A) Collinearity analysis between *A. avenae* and both *B. xylophilus* and *A. besseyi* reveals only a few conserved regions, suggesting a potentially independent evolutionary origin among them. **(B)** In contrast, *A. avenae* and *D. destructor* exhibits more extensive collinear blocks. Besides, a substantial number of collinear blocks were observed between *D. destructor*'s X chromosome and *A. avenae*'s Chromosome 2.

Supplemental_Fig_S16

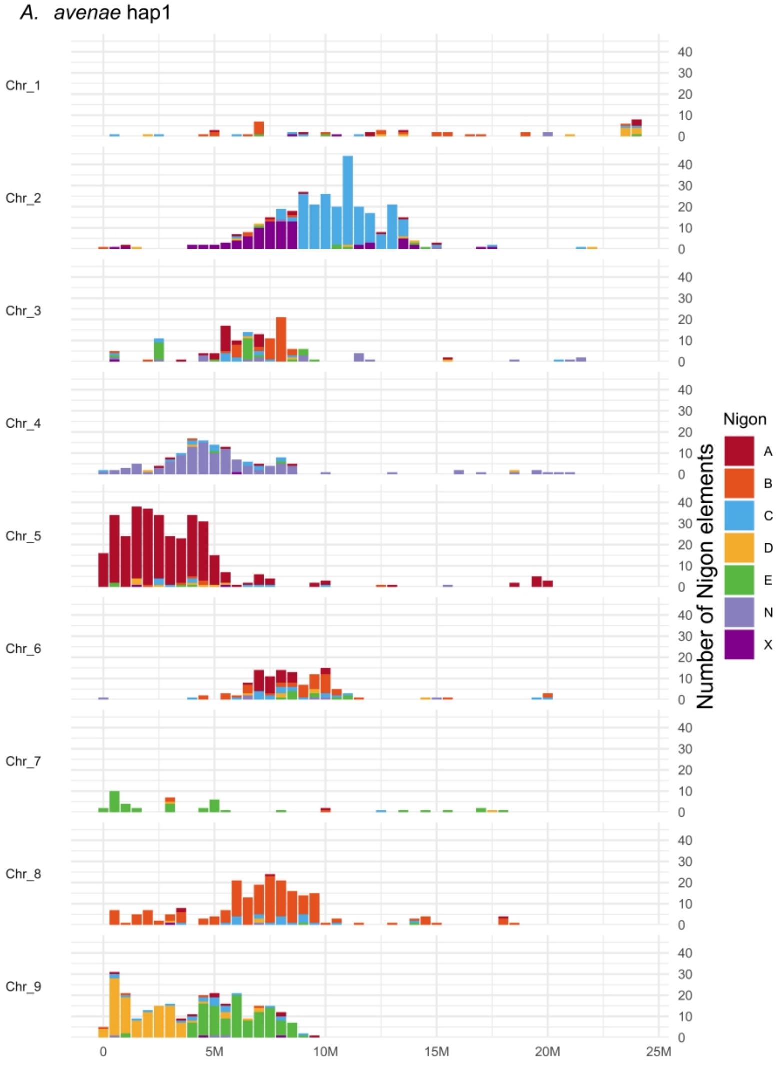

Fig. S16 Distribution of Nigon elements on each chromosome of *A. avenae* hap1. The distribution on hap2 is similar to that of hap1 and is therefore not shown here. Based on the distribution of Nigon X elements and the collinearity analysis between the X chromosome of *D. destructor* and chromosome 2 of *A. avenae*, we infer that Chromosome 2 of *A. avenae* is its X chromosome.

Supplemental_Fig_S17

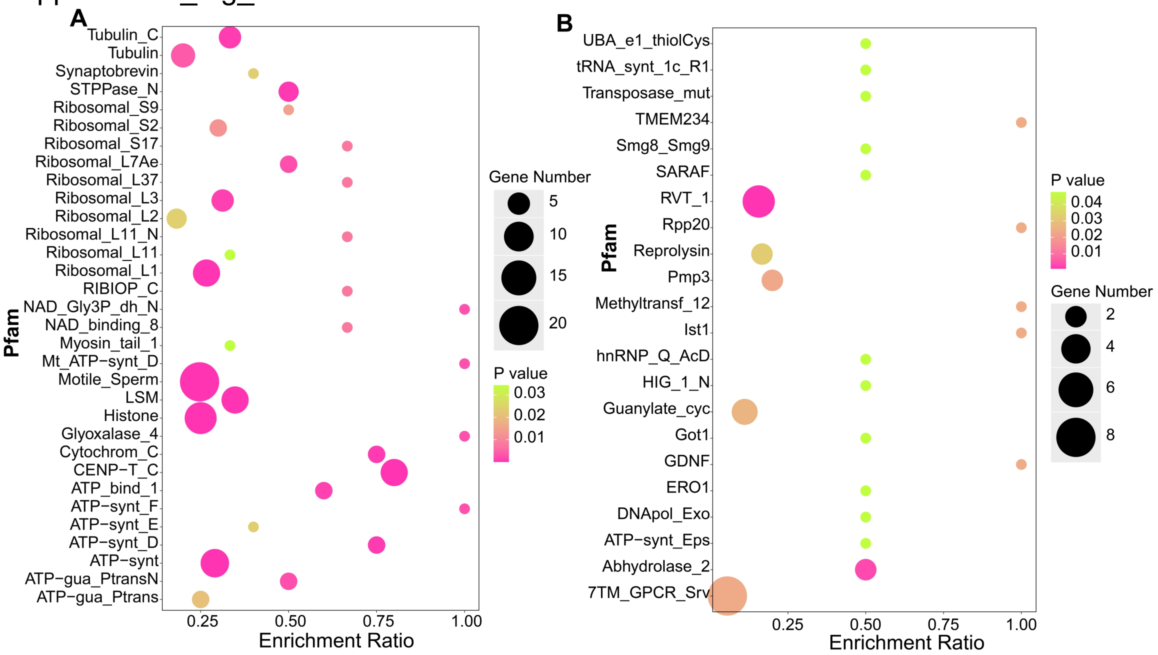

Fig. S17 The GO enrichment analysis of the different identical alleles. (A) The Pfam domain enrichment analysis for the alleles with completely identical sequences. **(B)** The Pfam domain enrichment analysis for the alleles with coverage less than 80%.

Supplemental_Fig_S18

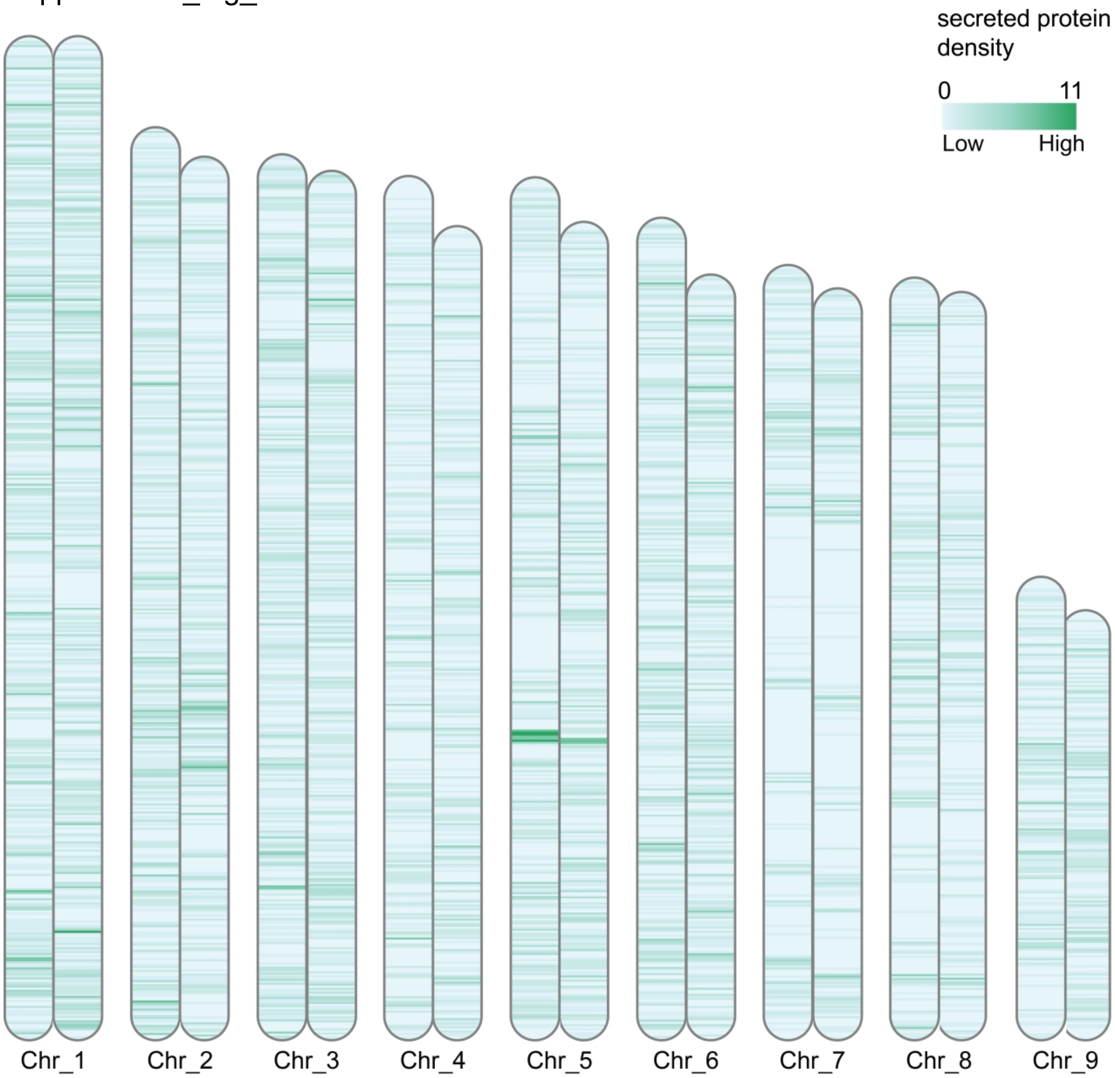

Fig. S18 The effector density on each chromosome of haplotype genome.

Supplemental_Fig_S19

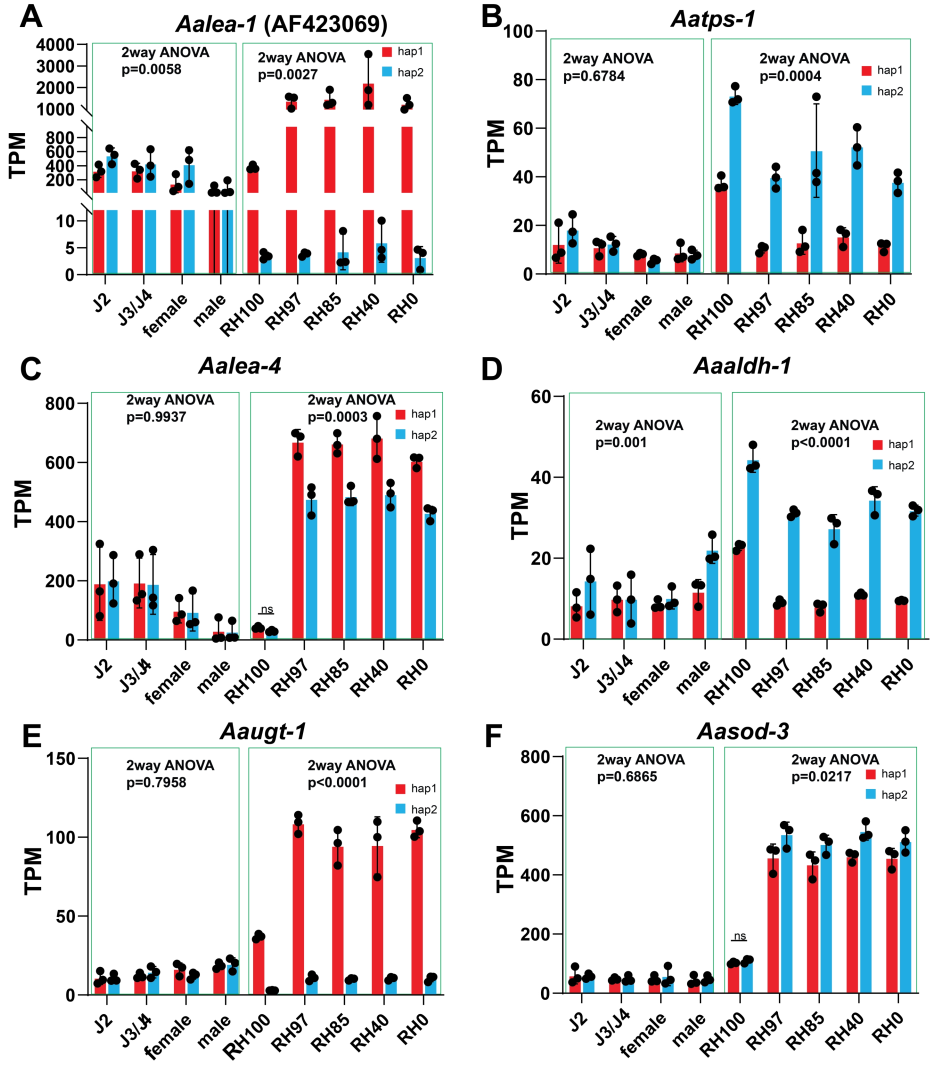

Fig. S19 Desiccation tolerance-related genes showed allelic unbalanced expression before and after desiccation stress. (**A** and **D**) There is a slight imbalance in allelic expression during normal growth, while a stronger imbalance occurs under drought stress. **(B, C, E,** and **F)** There is no imbalance in allelic expression during normal growth, while an allele imbalance expression occurs under desiccation stress. RH, Relative humidity.

Supplemental_Fig_S20

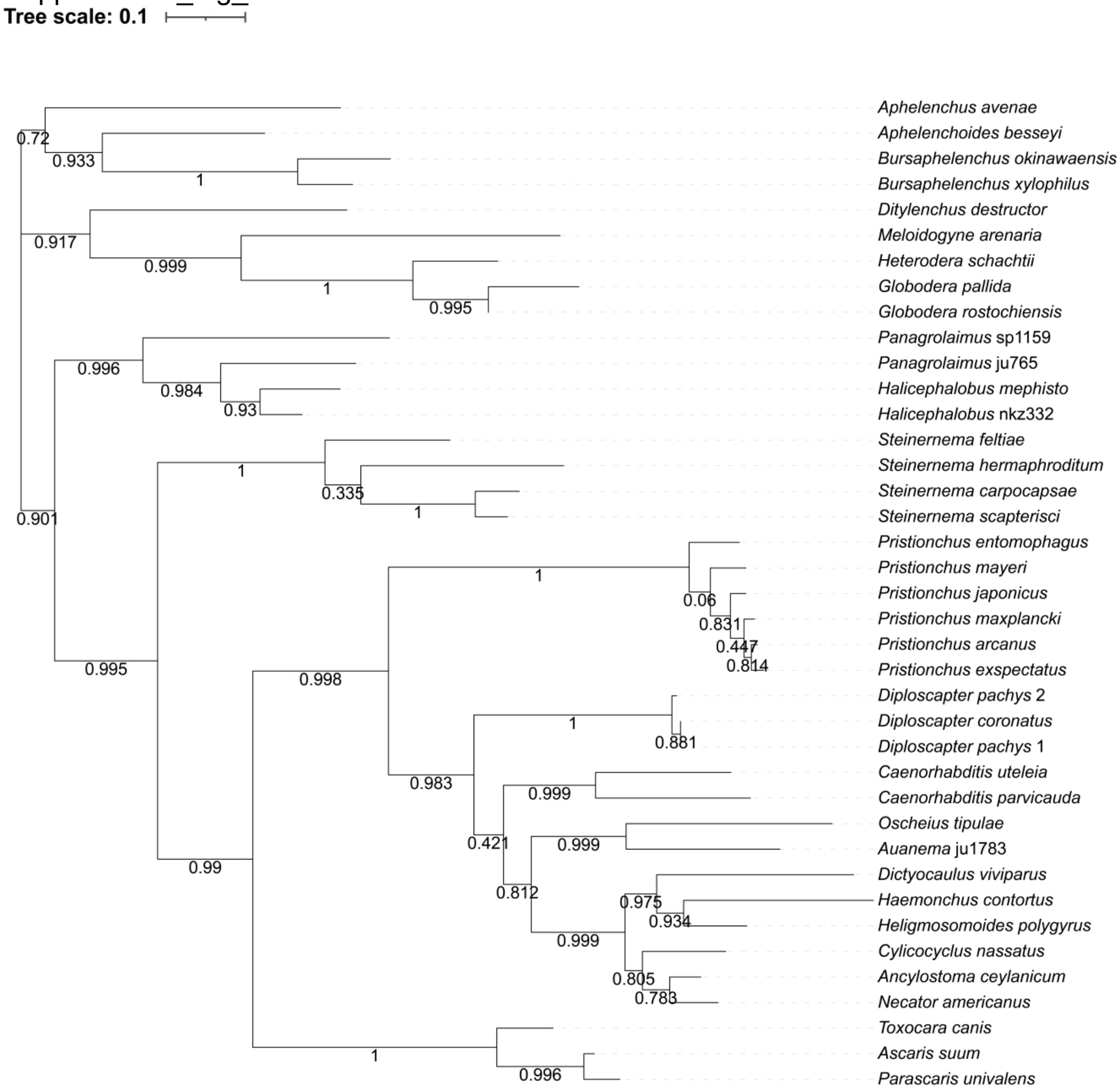

Fig. S20 Phylogenetic tree of *P5CS* genes among nematode species. *P5CS* genes exhibit high intrageneric conservation among different nematode groups.

Supplemental_Fig_S21

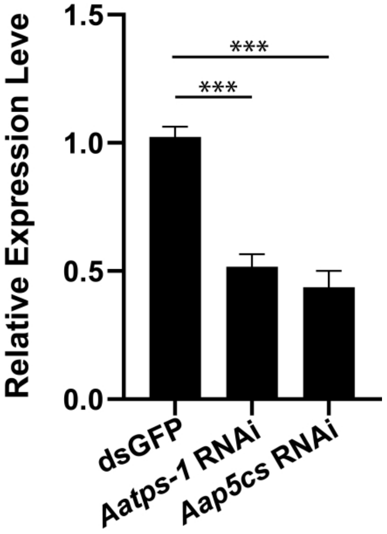

Fig. S21 The gene expression levels in *A. avenae* after RNAi were validated by RT-qPCR. Compared with the control ds*gfp* treatment, knockdown of both the *Aatps-1* and *Aap5cs* genes shows significant downregulation of these genes in *A. avenae*.

Supplemental_Fig_S22

***Aap5cs* RNAi**
0min desiccation

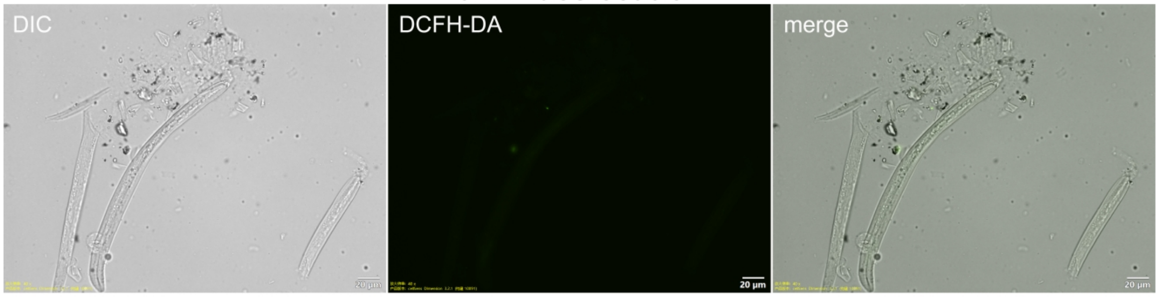

***Aatps-1* RNAi**
0min desiccation

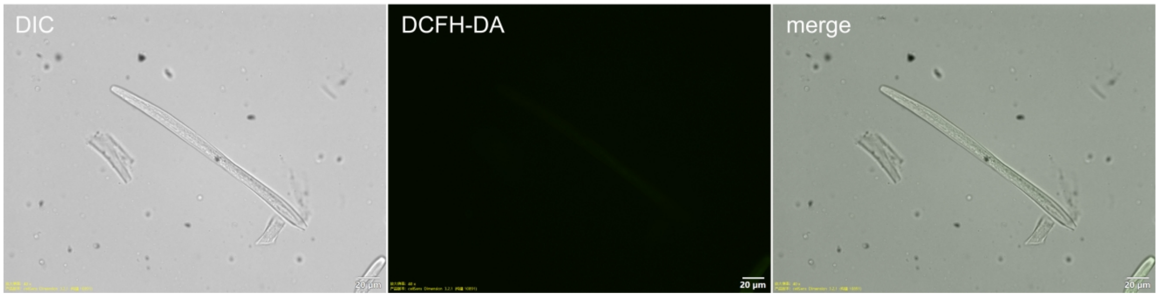

Fig. S22 ROS level detection of *Aap5cs* or *Aatps-1* dsRNA-treated *A. avenae* before dessication stress. After RNAi of *Aap5cs* and *Aatps-1*, DCFH-DA probe- based detection shows no ROS accumulation in the nematodes without desiccation stress.

Supplemental_Fig_S23

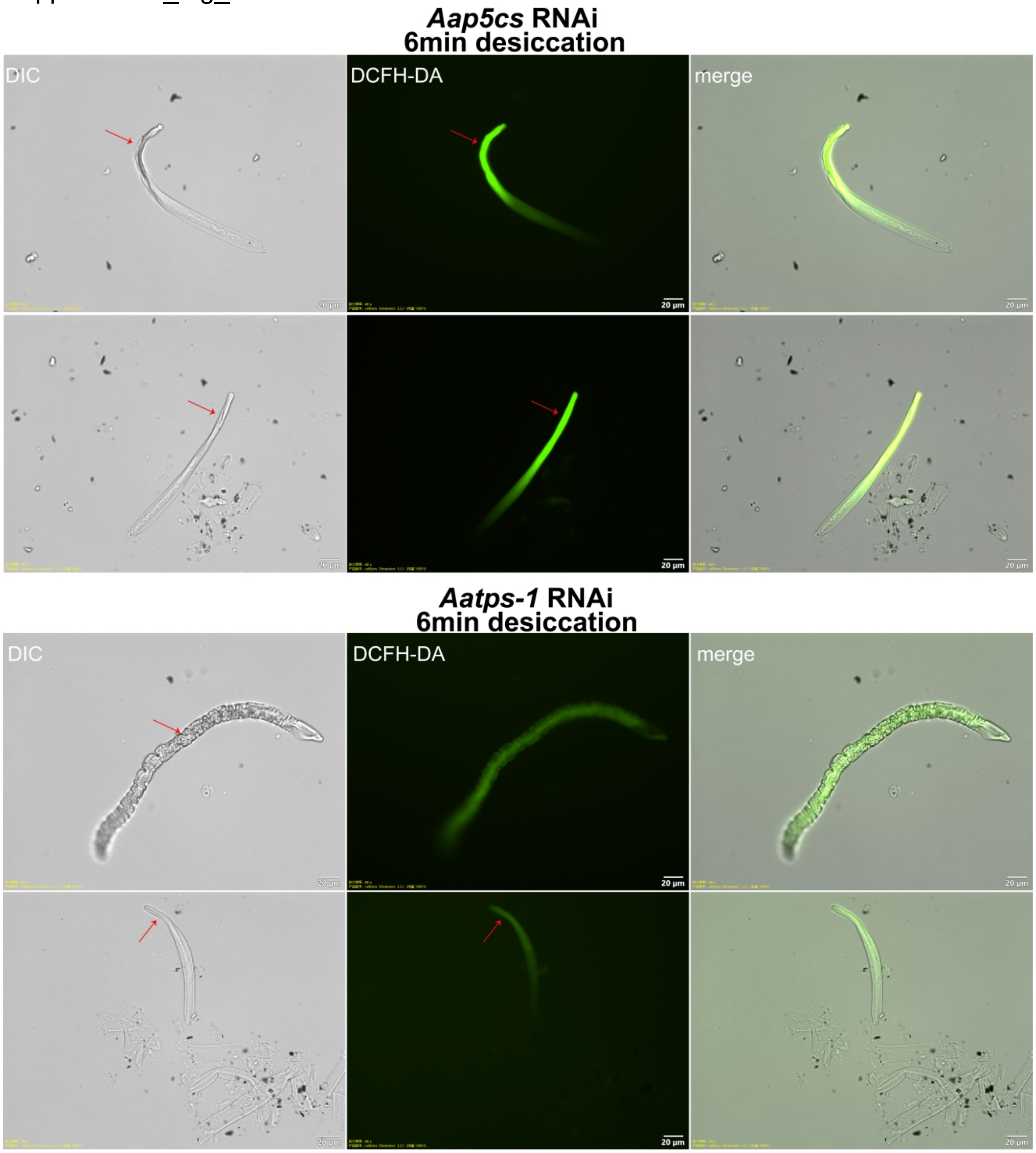

Fig. S23 The ROS level detection after 6min desiccation treatment of the *Aap5cs* and *Aatps-1* RNAi *A. avenae* nematodes. After treatment of 40% relative humidity for 6 minutes, DCFH-DA probe shows detection of ROS accumulation in the RNAi nematodes. *Aap5cs* RNAi nematodes have a large amount of ROS accumulation after 6 minutes of drying, with the dehydrated body region having more significant ROS accumulation. The red arrow indicates the dehydrated nematode region.

Supplemental_Fig_S24

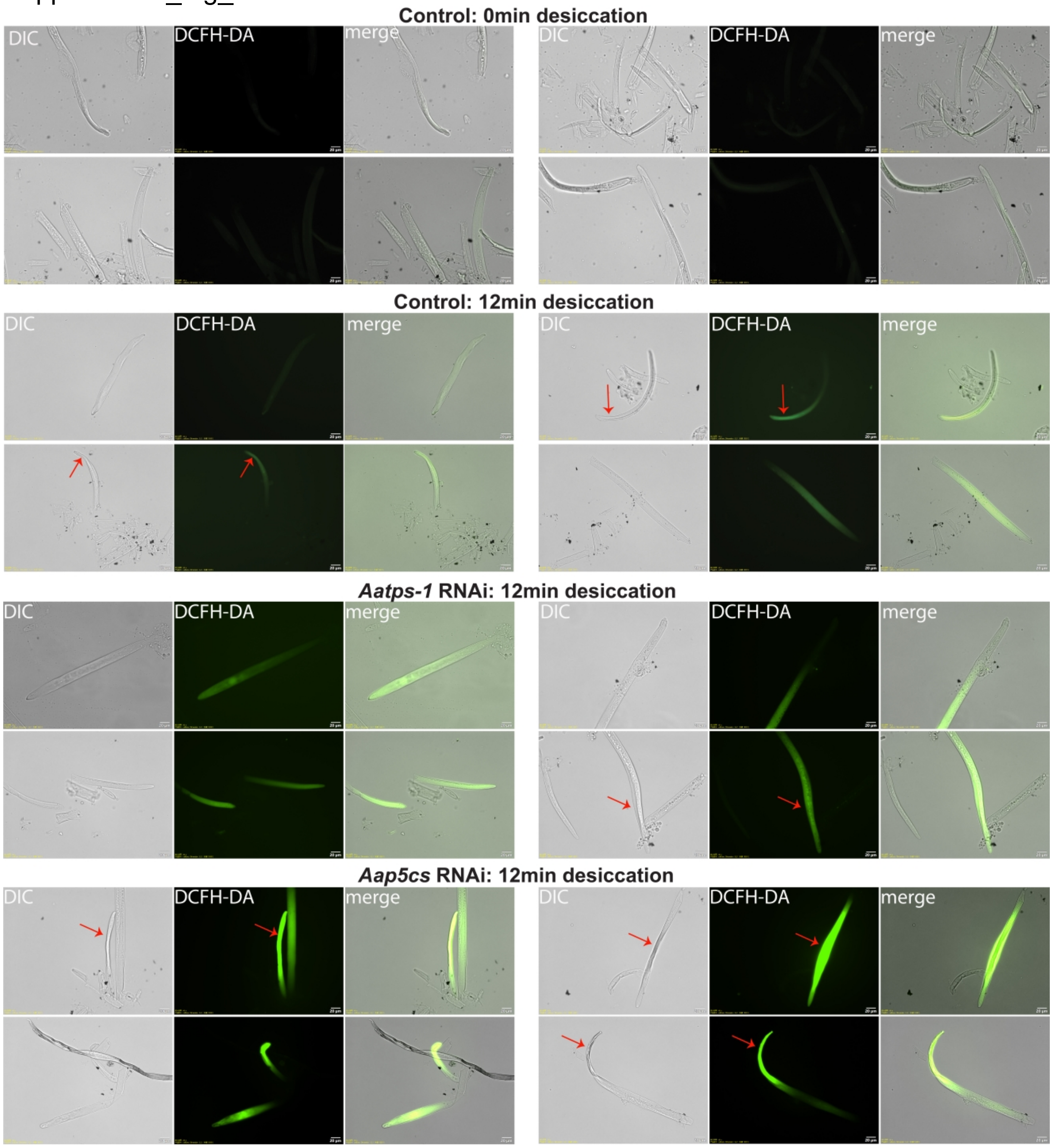

Fig. S24 The ROS level detection after 12min desiccation treatment of the *Aap5cs* and *Aatps-1* RNAi *A. avenae* nematodes. The red arrow indicates the dehydrated region in the nematode. Bar = 20 μm.
